# Supplementary material for: Sequential Binding of MEIS1 and NKX2-5 on the Popdc2 Gene: A Mechanism for Spatiotemporal Regulation of Enhancers during Cardiogenesis
Source: Cell Rep. 2015 Sep 24;13(1):183–95. doi: 10.1016/j.celrep.2015.08.065 (PMC4597108; doi:10.1016/j.celrep.2015.08.065)
Supplement: Document S1. Supplemental Experimental Procedures, Figures S1–S5, Tables S1 and S2 [file mmc1.pdf]

Cell Reports

Supplemental Information

Sequential Binding of MEIS1 and NKX2-5 on the Popdc2 Gene: A Mechanism for Spatiotemporal Regulation of Enhancers during Cardiogenesis

Laurent Dupays, Catherine Shang, Robert Wilson, Surendra Kotecha, Sophie Wood, Norma Towers, and Timothy Mohun

Supplemental Experimental Procedures

Chromatin Immunoprecipitation assay

Chromatin was prepared from 20-25 mouse embryonic hearts at stage E11.5. Tissue was fixed for 3 hours at room temperature in buffer (50mM HEPES pH7.9, 1mM EDTA, 1mM EGTA, 100mM NaCl, 0.07% butryric acid) containing 1.8% formaldehyde. Heart tissue was homogenised using an Ultra-Turrax, T25 basic (IKA-Werke), and pelleted at low speed. Samples were washed twice with ice-cold PBS with freshly added EDTA-free protease inhibitors (PI) (Roche). Samples were resuspended in 1.5ml lysis buffer (25mM Tris pH 7.5, 150mM NaCl, 1% Triton X100, 1% SDS, 2.5mM Sodium deoxycholate, PI) and transferred to RNase-free non-stick microfuge tubes (Ambion). Samples sonicated for 15 x 30 seconds on ice using a Branson Digital Sonicator with a 2.5mm stepped probe tip. Samples were spun at 15K using a bench top centrifuge for 15 minutes at 4oC. The supernatant was then pre-blocked with protein A/G sepharose (Perbio), pre-treated with BSA (Biorad) and Poly (dI-dC)-poly (dI-dC) (GE Healthcare). A proportion of the sample was kept at this stage as an input control. 150ml of chromatin was diluted 10 fold in ChIP dilution buffer (16.7mM Tris pH7.5, 0.01% SDS, 1.1% Triton, 1.2mM EDTA, 167mM NaCl) and 4mg of the antibody was added and incubated overnight at 4°C with rotation. The antibody:protein:DNA complexes were captured using pre-blocked protein A/G sepharose for 2 hours rotating at 4°C. The beads were then spun down and washed twice in wash buffer A (10mM HEPES pH 7.6, 1mM EDTA, 0.5mM EGTA, 0.25% Triton X100)

and twice with wash buffer B (10mM HEPES pH7.6, 200mM NaCl, 1mM EDTA, 0.5mM EGTA, 0.01% Triton X100. After extensive washing the beads were resuspended in TE and cross-links reversed overnight at 65°C. Samples were adjusted to 0.1% SDS, digested with 10mg Proteinase K (Roche) at 50°C for 3 hours and extracted twice with phenol/chloroform. Samples were ethanol precipitated with glycogen carrier (Ambion), and resuspended in 100ml TE. Antibodies used in the ChIP assays were NKX-2.5 (N-19) sc-8697 (Santa Cruz) and MEIS1 (Abcam, ab19867).

For experiment at E9.5, 40 to 50 embryos were dissected and AHF, heart were separately collected and processed as described previously.

Quantitative Real-Time PCR analysis

The fold enrichment of genomic targets in the immunoprecipitated DNA was determined by Real-Time quantitative PCR (qPCR) using the ABI PRISM 7000 sequence detection system (Applied Biosystems). Regions were considered enriched in binding if they displayed greater than a cut-off value of 1.5-fold. Primers were designed using Primer Express software (Applied Biosystems) and are available on demand. Relative fold enrichment was determined by normalising the Ct values for input (ΔCt) by subtracting the average Ct value of input from average Ct value of IP (ΔCt = $averageCt_{ChIP}$- $averageCt_{input}$). Enrichment was then calculated relative to a negative control (promoter region of eye specific g-crystallin gene) using the formula 2-($\Delta Ct_{[target]}$-$\Delta Ct_{[gCrystallin]}$). Fold

enrichment was calculated by dividing the enrichment values from the antibody-specific- ChIP by the enrichment values obtained in the background (no antibody) control samples. All qPCR were performed on at least four independent ChIPs.

Sequencing and peak calling

Libraries were prepared according to Illumina's instructions accompanying the DNA Sample Kit (Part# 0801-0303) with 10ng of immunoprecipitated DNA or input. Samples were sequenced on the Genome Analyser IIx platform and initial analysis was carried out using Illumina's CASAVA software. ChIP-seq reads were aligned to the mm9 genome assembly using BOWTIE. Peak calling was done using MACS1.4 (Stringency: p-value = 1x10-14) on the two independent experiments (s1 and s4) using a background experiment consisting of the input DNA. 2610 peaks were identified as the intersection between peaks called in the s1 and s4 experiments. Gene to peak assignment was achieved using GREAT (Two nearest genes association rule) (McLean et al., 2010).

Cell culture and stable transfections

HL-1 cardiac myocytes were maintained as previously reported (Claycomb et al., 1998). Briefly, HL-1 cells were grown in Claycomb medium (Sigma-Aldrich) supplemented with 10% fetal bovine serum (Sigma-Aldrich), 0.1 mM norepinephrine (Sigma-Aldrich), 2 mM L-glutamine (Sigma-Aldrich), and penicillin/streptomycin (Invitrogen) in a humidified 5% CO_2 incubator at 37°C on

fibronectin-coated flasks. After several passages, cells were transfected with 1 mg of shRNA against *Nkx2-5* (SureSilencing shRNA Plasmid for Mouse *Nkx2-5*, KM04476P, Qiagen) per plate, (or with comparable amounts of a control) using Fugene reagent (Roche). On the second day after transfection, fresh growth media supplemented with 1 μg/ml puromycin (Invitrogen) as the selection agent was added. Selection was allowed to proceed for 14 days, and the resulting colonies were harvested and maintained in the selection medium.

Immunoblotting

Cells were homogenized in 0.5 ml of RIPA buffer (20 mM Tris-HCl, pH 8.0; 150 mM NaCl; 0.1% SDS; 1% NP-40; 0.5% Sodium deoxycholate; and complete protease inhibitors cocktail; Roche). Protein concentration was determined with the Pierce BCA protein assay kit. 20 mg of protein extracts were separated on SDS-polyacrylamide gels and transferred to nitrocellulose membranes. Membranes were probed with an antibody against NKX2-5 (1:500) and subsequently with a goat anti-mouse IgG-HRP (1:2,000 dilution, sc-2005; Santa Cruz Bio- technology Inc.).

Dual Luciferase Reporter Assay

Denovo were synthesize in vitro and clone into pGL3 promoter vector (Promega).

Tnni2-Tnnt3 481bp enhancer was amplified by PCR from genomic DNA with the primers forward: TGCTACTCTTGGGGCTGACTTTAT and reverse GGACACCTGCCTGCTTTTTCTTC and cloned into pGL3 promoter vector (Promega). Tnni2-Tnnt3 mutated enhancer was obtain using QuickChange II site directed mutagenesis kit (Agilant Technologies) with the primers Forward: 5' CAGTGAAAACCTCTTAAGCCTCGTCAGGACAGAAGTGGC 3' and Reverse: 5' GCCACTTCTGTCCTGACGAGGCTTAAGAGGTTTTCACTG 3'.

Meis1 and Nkx2-5 cDNA cloned into pcDNA3 were transfected accordingly to the quantity indicate on the figures. pcDNA3 alone was used as control.

Transient transfections were performed in HL-1 cells or 3T3 cells using Lipofectamine 2000 (Promega) according to the manufacturer's instructions. Total cell lysates were prepared 48 hr posttransfection and luciferase activity was assessed using the Promega Dual Luciferase Reporter kit (Promega) according to the manufacturer's protocol. Results were normalised to a renilla transfection control.

Claycomb, W.C., Lanson, N.A., Jr., Stallworth, B.S., Egeland, D.B., Delcarpio, J.B., Bahinski, A., and Izzo, N.J., Jr. (1998). HL-1 cells: a cardiac muscle cell line that contracts and retains phenotypic characteristics of the adult cardiomyocyte. Proc Natl Acad Sci U S A 95, 2979-2984.

McLean, C.Y., Bristor, D., Hiller, M., Clarke, S.L., Schaar, B.T., Lowe, C.B., Wenger, A.M., and Bejerano, G. (2010). GREAT improves functional interpretation of cis-regulatory regions. Nat Biotechnol 28, 495-501.

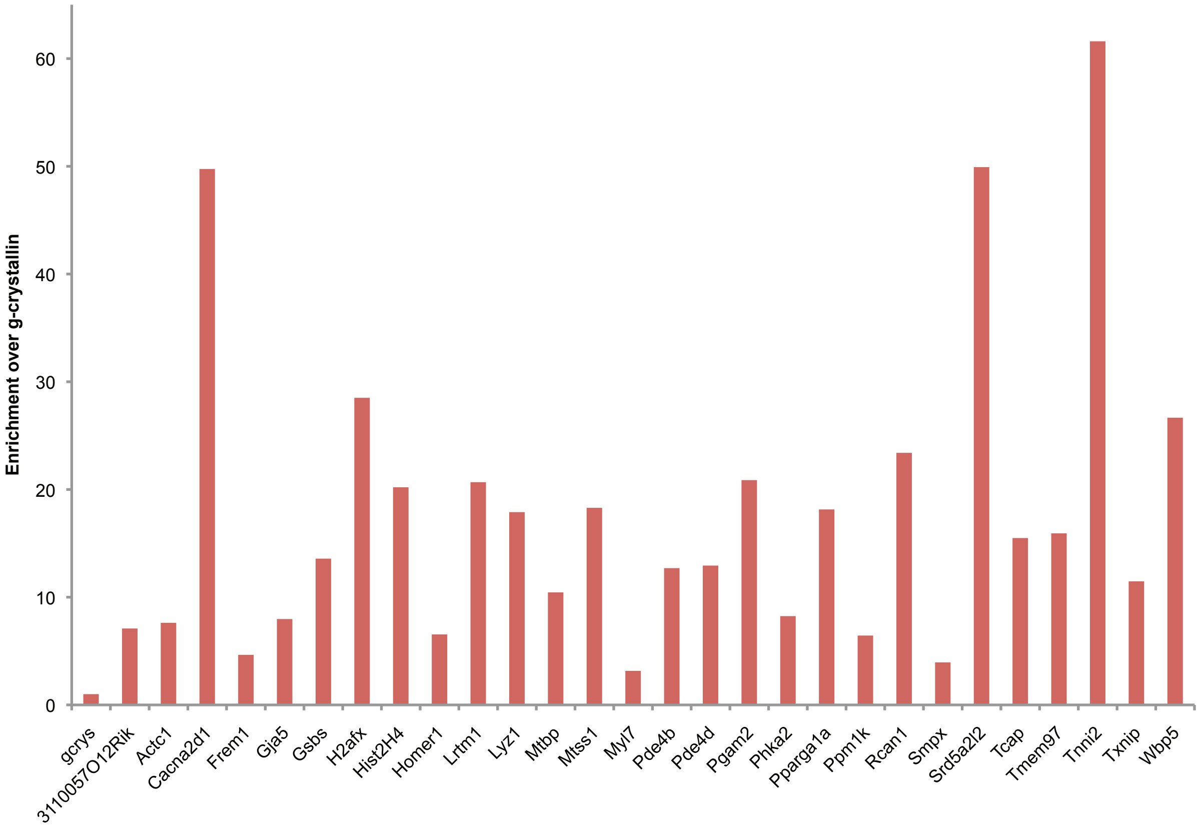

Figure S1. Validation of NKX2-5 enriched regions by ChIP-qPCR, Related to figure 1. Chromatin immunoprecipitation followed by quantitative PCR showing relative enrichment of the chosen enriched region for NKX2-5 binding over a negative control region in the gamma-crystallin gene using chromatin from E11.5 embryonic hearts.

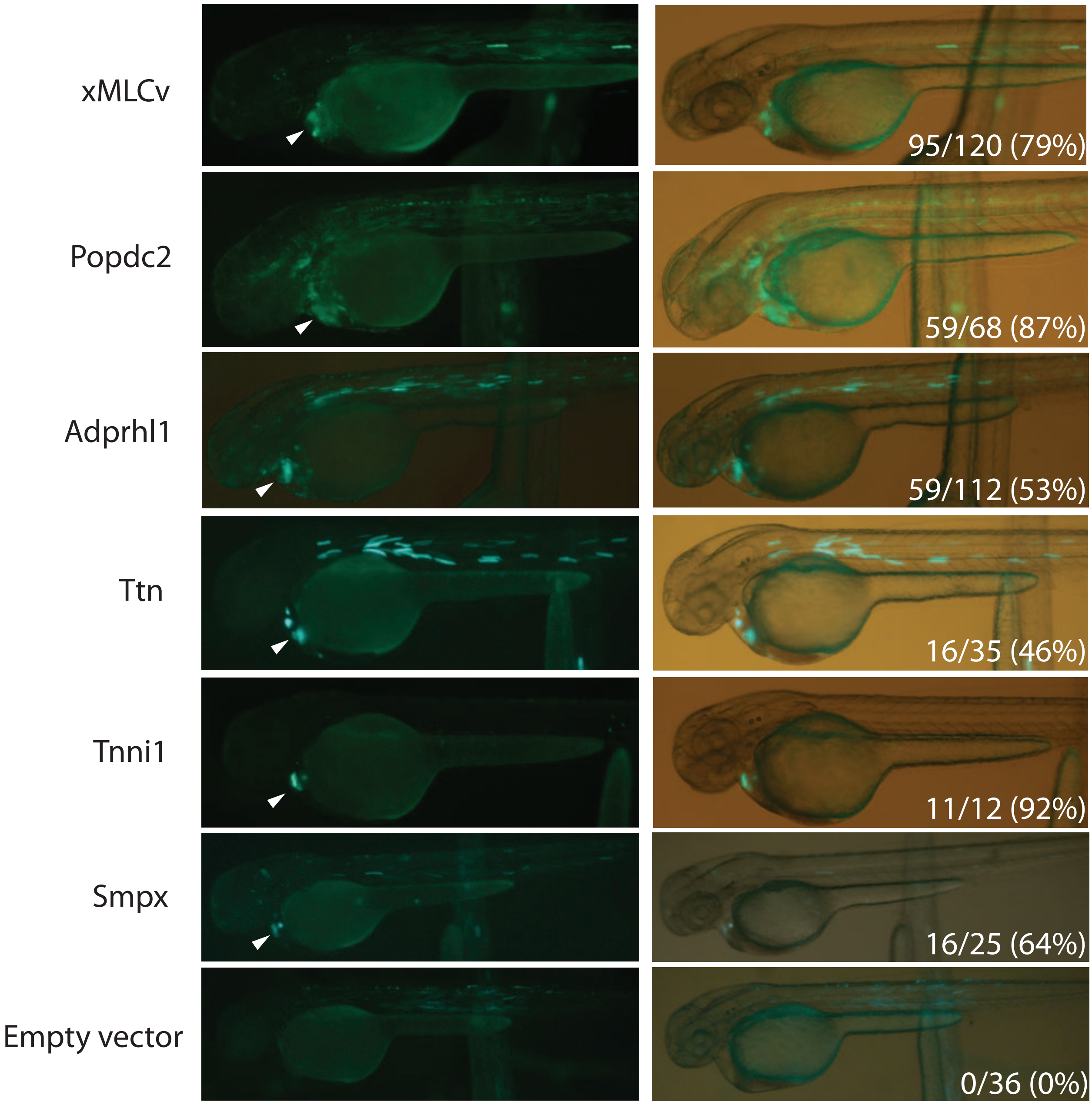

Figure S2. Validation of NKX2-5 enriched regions as enhancers, Related to Figure 1. Five different NKX2-5-binding regions (associated with the genes Popdc2, Adprhl1, Ttn, Tnni1 and Smpx) were tested for their ability to drive cardiac expression (white arrowheads). Left side view of a representative transgenic zebrafish embryo at 48 hours post-fertilization (Left panel: GFP filter; right panel: overlay with bright field.). Percentage of cardiac expressing embryos in the GFP positive pool is indicated. xMLC is used as a positive cardiac enhancer. Note that an empty vector shows no detectable GFP expression.

A

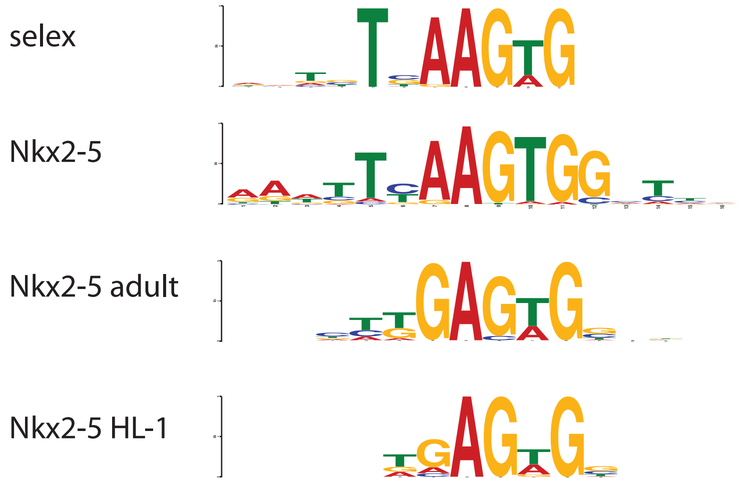

B

selex	TGCCCAGTCAAGTGTTCTTGA
denovo	ACAAAGTGGTGACAGGATCC
denovo m1	ACAAAGCCTCGACAGGATCC
denovo m2	ACAAAGTGGTCCTCGGATCC

Figure S3. ChIP-seq motif discovery comparison, Related to Figure 1. (A) Comparison of the selex NKX2-5 DNA binding motif with published binding motifs "NKX2-5" (Uniprobe), "NKX2-5 adult" (van den Boogaard et al., 2012) and "NKX2-5 HL-1" (He et al., 2011). (B) Sequence of the different probes used in the EMSA assay.

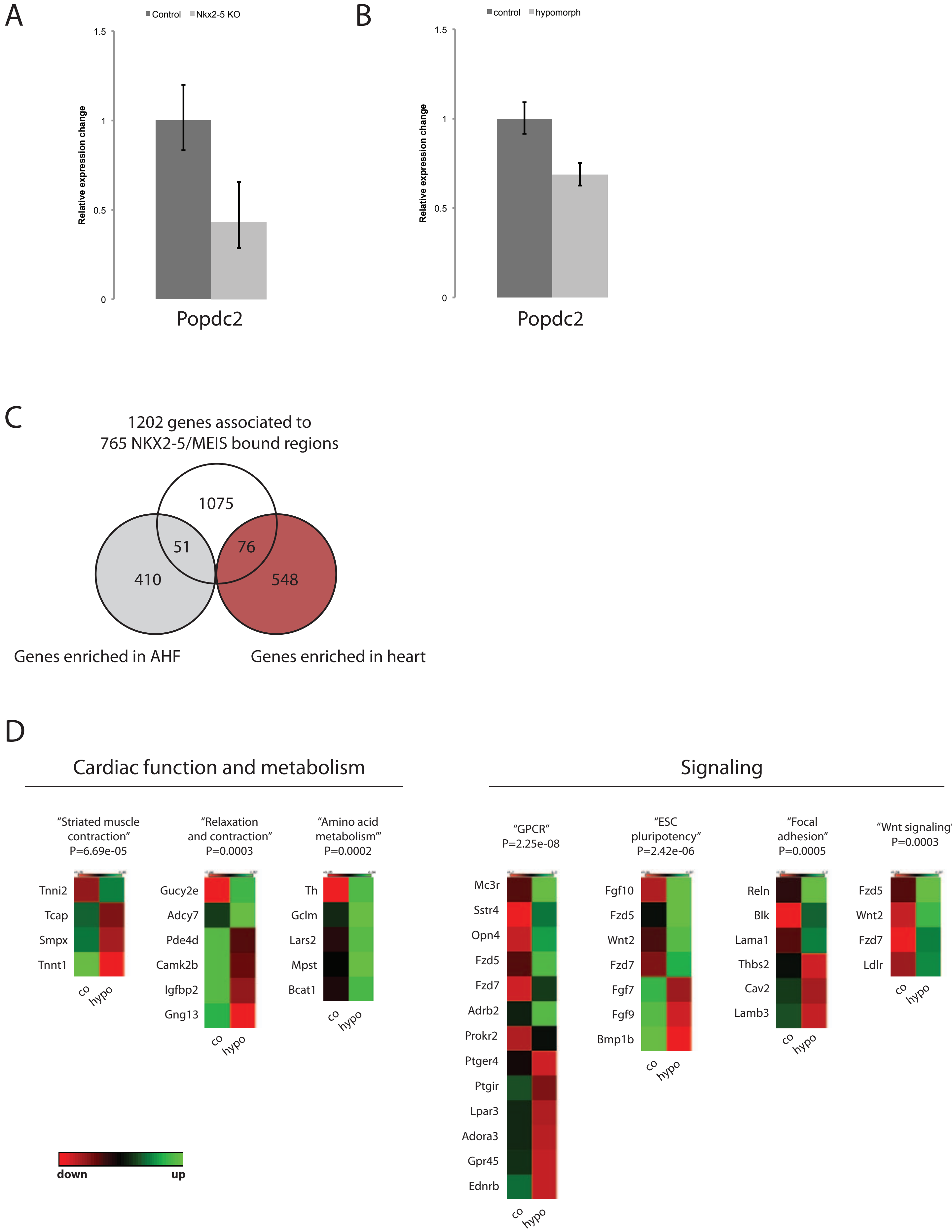

Figure S4. Enriched pathways, Related to Figure 5. Panels (A) and (B): Relative quantitative RT-PCR for Popdc2 mRNA expression in isolated embryo hearts. (A) Control and NKX2-5 heart knock out (Nkx2-5-gfp/Nkx2-5-gfp) at E9; (B) control and NKX2-5 hypomorph (Nkx2-5-IRES-cre/Nkx2-5-gfp) at E11.5. Panel C: Venn diagram showing the number of genes associated with an NKX2-5/MEIS bound region that are also enriched in AHF (51 genes) or heart (76 genes) according to Domian et al., 2009. Panel (D): The top seven Wikipathways enriched in the direct targets list. For each pathway, a p value is indicated. A heat map for each gene is shown, using data from RNA-seq with the NKX2-5 mutant hypomorph.

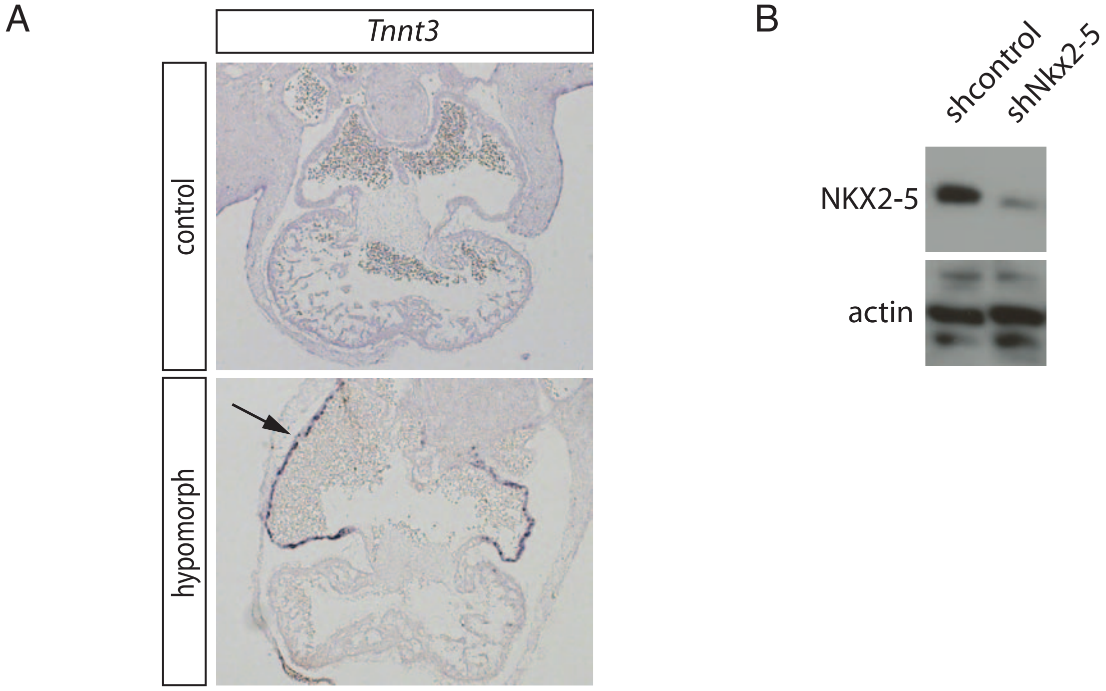

Figure S5. Tnnt3 expression at E11.5, Related to Figure 6. In situ hybridization on sections from E11.5 control and hypomorphic hearts, showing misexpression of Tnnt3 RNA in the atria (black arrow). (B) Western-blot showing downregulation of NKX2-5 protein expression in HL-1 cells transfected with a shRNA against Nkx2-5.

		Total number of peaks	Number of peaks overlapping With Nkx2-5 set	%	Median randomized	%	Fold enrichement
This study	**Nkx2-5 E11.5**	2610					
Blow et al., 2010.	**p300 E11.5**	3597	392	15.02%	5	0.19%	78.4
Shen et al., 2011.	**Tbx20 adult**	4012	354	13.56%	4	0.15%	88.5
Van Den Boogaard et al., 2012.	**Gata4 adult**	1756	231	8.85%	1	0.04%	231.0
	**Nkx2-5 adult**	6705	635	24.33%	6	0.23%	105.8
	**Tbx3 adult**	13242	416	15.94%	11	0.42%	37.8
He et al., 2011.	**Gata4 HL_1**	16753	803	30.77%	17	0.65%	47.2
	**Mef2a HL-1**	1337	104	3.98%	1	0.04%	104.0
	**Nkx2-5 HL-1**	20573	638	24.44%	20	0.77%	31.9
	**Srf HL-1**	23806	506	19.39%	23	0.88%	22.0
	**Tbx5 HL-1**	55872	933	35.75%	59	2.26%	15.8
	**p300 HL-1**	1491	264	10.11%	1	0.04%	264.0
Amin et al., 2015.	**Meis 1st BA**	64407	765	29.31%	15	0.57%	51.0
	**Meis 2nd BA**	62628	787	30.15%	16	0.61%	49.2

Table S1. Overlap between E11.5 NKX2-5 enriched regions (this study) and previously published data for various cardiac transcription factors, Related to Figure 1. The number of regions enriched and the number of overlapping regions with each of these factors and NKX2-5 are presented. Median randomized region number represents the median of the distribution of randomized intersections (10.000 iterations) between each set and randomly generated set similar to NKX2-5. Fold enrichment represents the actual count divided by the median for each factor and can be considered as a score.

	+/+	gfp/+	cre/+	gfp / cre	Total number of embryos
E8.5	23.1%	19.2%	30.8%	26.9%	26
E9.5	27.8%	21.1%	22.2%	28.9%	90
E11.5	24.3%	27.8%	24.3%	23.6%	403
E12.5	31.0%	17.2%	24.1%	27.6%	58
E13.5	25.6%	23.1%	23.1%	28.2%	39
E14.5	22.6%	38.7%	21.0%	17.7%	62
Weaning	31.8%	36.4%	31.8%	0.0%	298

Table S2. Mendelian analysis, Related to Figure 5. Number of different embryos collected prenatally and postnatally using the hypomorphic transgenic line.
