# Supplementary material for: Sequential Binding of MEIS1 and NKX2-5 on the Popdc2 Gene: A Mechanism for Spatiotemporal Regulation of Enhancers during Cardiogenesis
Source: Cell Rep. 2015 Sep 24;13(1):183–95. doi: 10.1016/j.celrep.2015.08.065 (PMC4597108; doi:10.1016/j.celrep.2015.08.065)
Supplement: Data S1. Genome Coordinates for Common Peaks in Both Experiments, Related to Figure 1 [file mmc2.pdf]

Dip2b

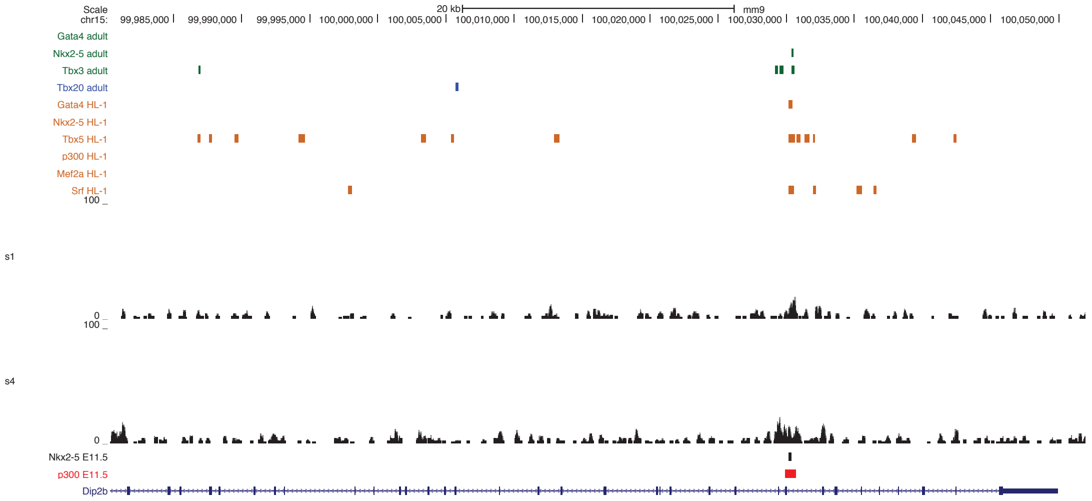

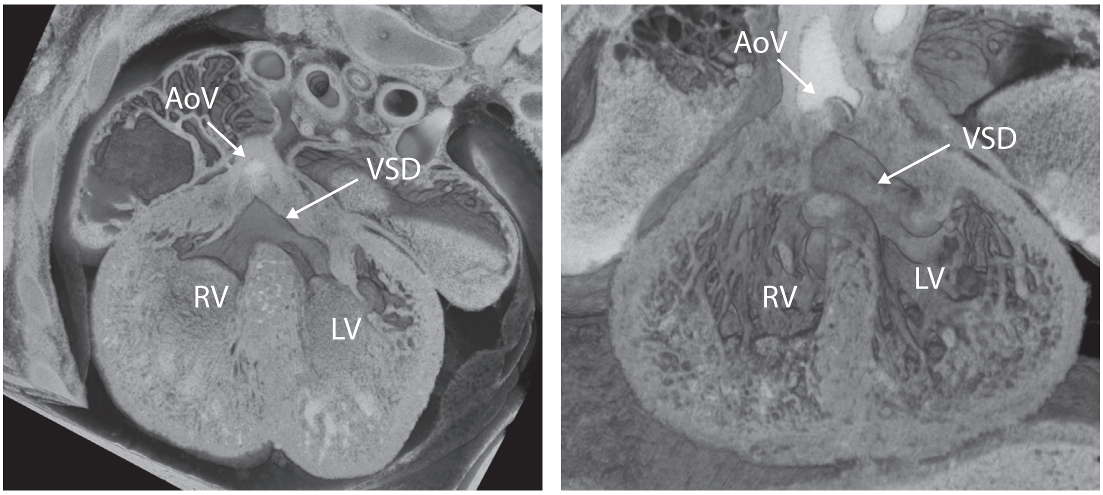

Data S2. Description of the locus and phenotype associated with the 6 genes targets of NKX2-5 present in the mouse phenotype database, Related to FIgure1. Above and underneath the reads profile of both experiments reported in this paper at E11.5 are presented the regions found statistically enriched in our experiment (black), for P300 at E11.5 in heart (red from Blow et al., 2010), for GATA4, NKX2-5, TBX3 and TBX20 in adult heart (green from Van Den Boogaard et al., 2012 and blue from Shen et al., 2011) and for GATA4, NKX2-5, TBX5, P300, MEF2A and SRF in HL-1 cells (orange from He et al., 2011). s1 and s4 are representing the two experiments carried out. 3D reconstruction of image data for the mutant hearts obtained using high resolution episcopic microscopy (http://embryoimaging.org) reveals the severity of the heart defects detected at E14.5. All six mutants show large ventricular septal defects (VSD) between the cushions of the atrioventricular junction and the crest of the muscular ventricular septum. In addition, Mks1 shows multiple additional VSD's through more apical (muscular) regions of the interventricular septum. Dot1l, Mks1, Mysm1 and Pds5b all show abnormally thin ventricular walls (white arrowhead) with an apparent increase in the extent of the ventricular trabecular network (asterisk). All mutants show aberrant connection of the great vessels to the heart chambers. Dip2b, Mysm1 and Spnb2 show an overriding aorta (OA) whilst Spnb2, Mks1 and Dot1l all connection of both the aortic and pulmonary roots to the right ventricle (DORV). AoV: aortic valve; LV: left ventricle; PT: pulmonary trunk; PV: pulmonary valve; RV: right ventricle; VSD: ventricular septal defect.

Dot1l

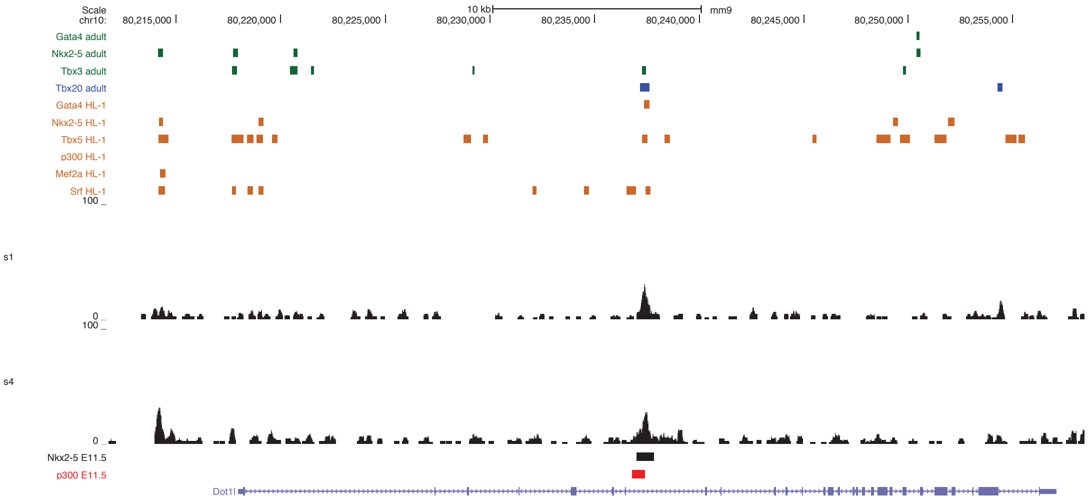

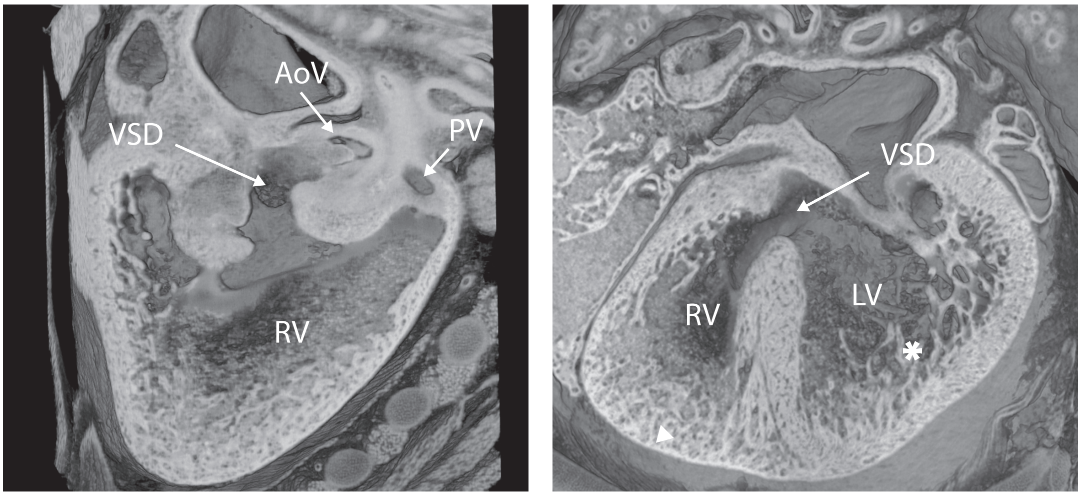

Data S2.

Mks1

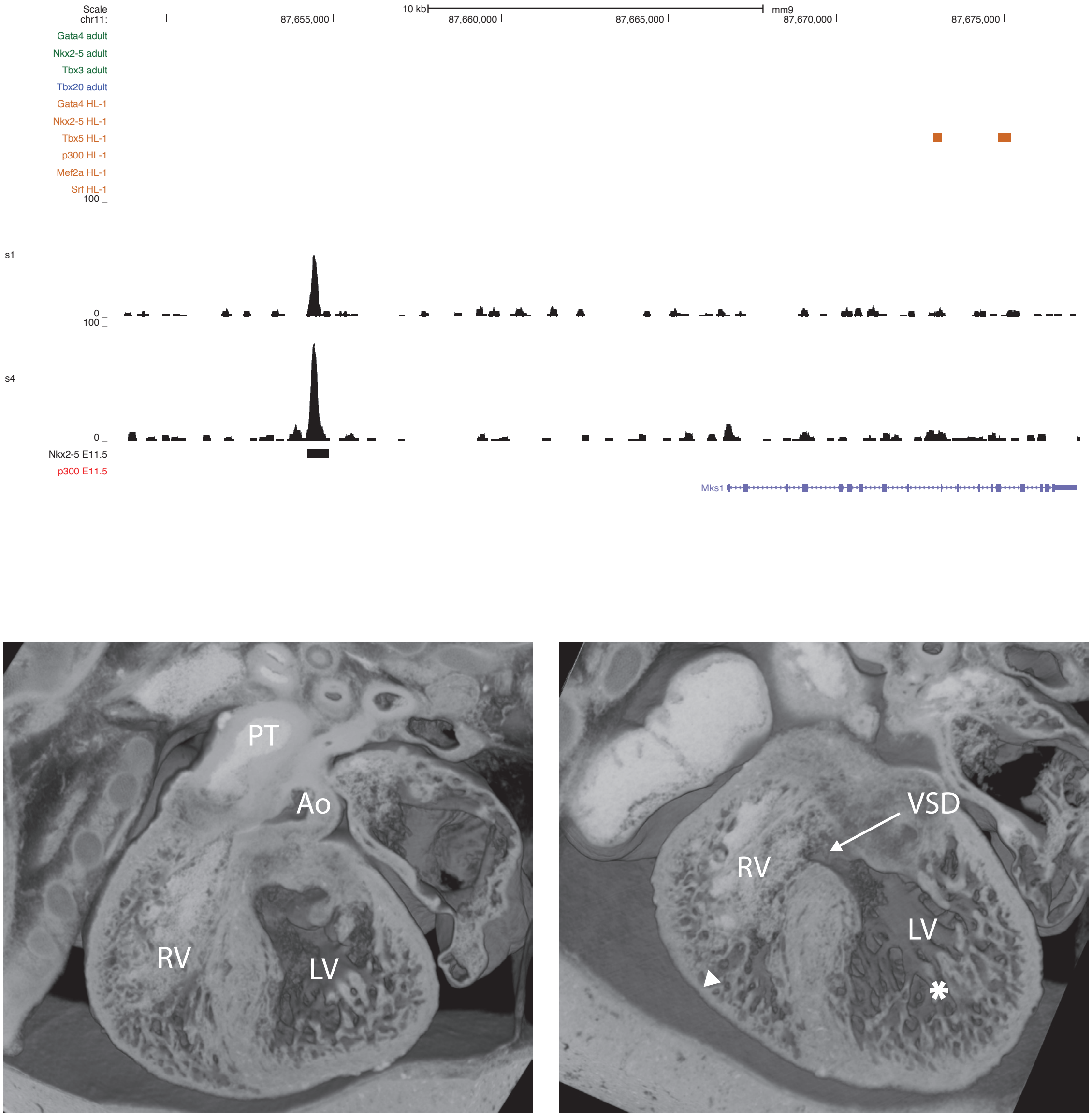
Scale
chr11:
10 kb
mm9
87,655,000
87,660,000
87,665,000
87,670,000
87,675,000
Gata4 adult
Nkx2-5 adult
Tbx3 adult
Tbx20 adult
Gata4 HL-1
Nkx2-5 HL-1
Tbx5 HL-1
p300 HL-1
Mef2a HL-1
Srf HL-1
100 _
s1
0 _
100 _
s4
0 _
Nkx2-5 E11.5
p300 E11.5
Mks1
PT
Ao
RV
LV
VSD
RV
LV
*

Data S2.

Mysm1

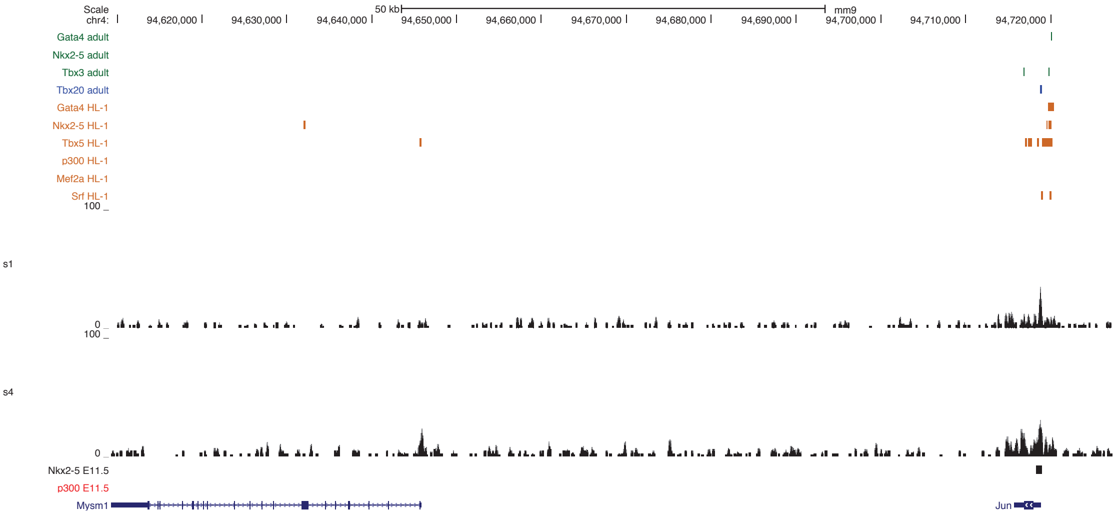

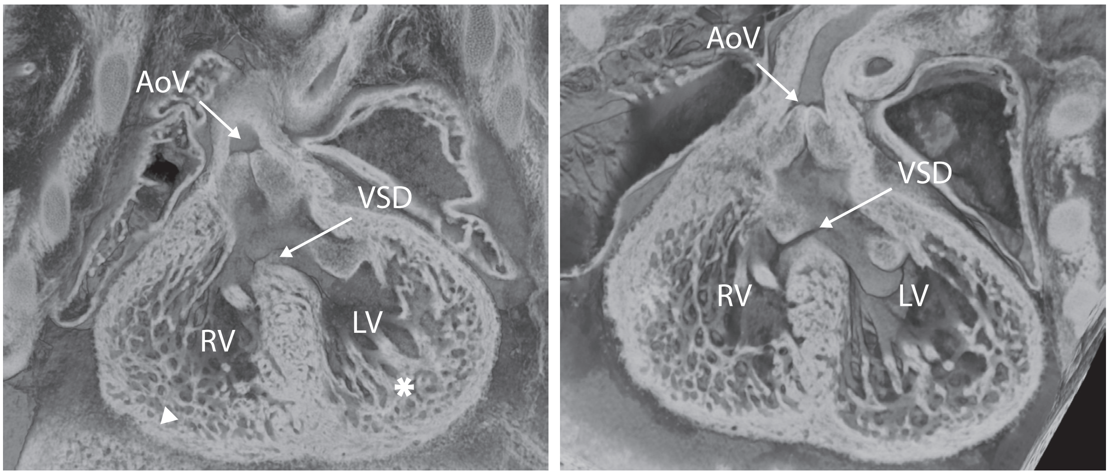

Data S2.

Pds5b

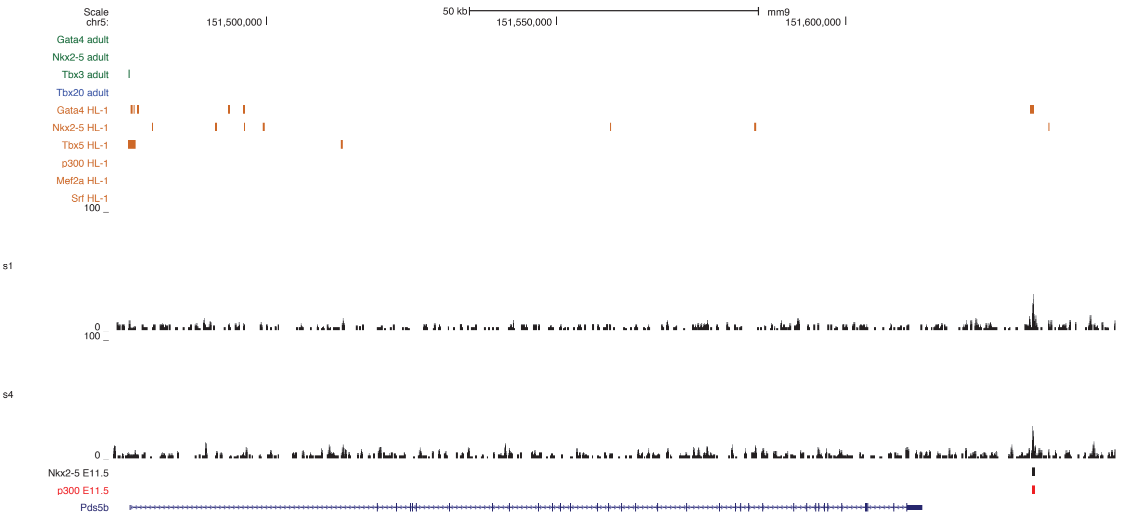

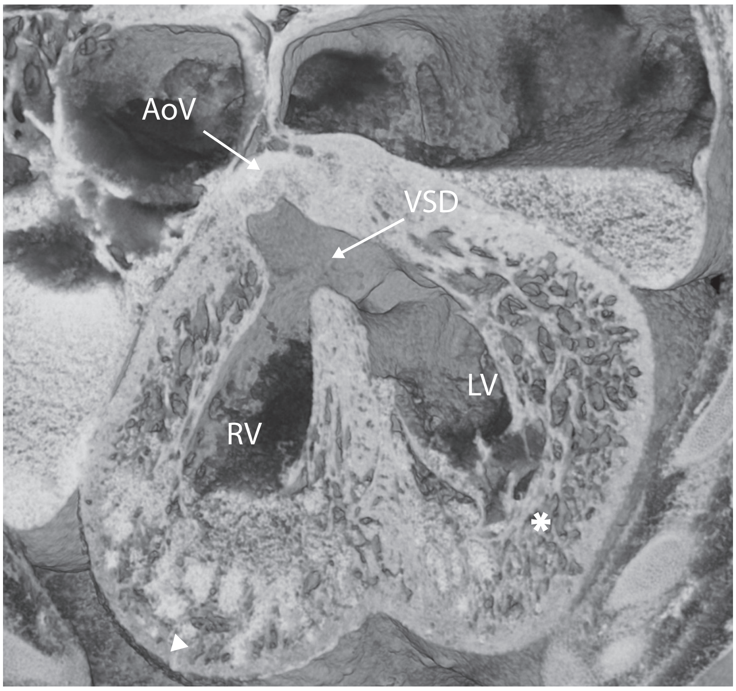

Data S2.

Spnb2

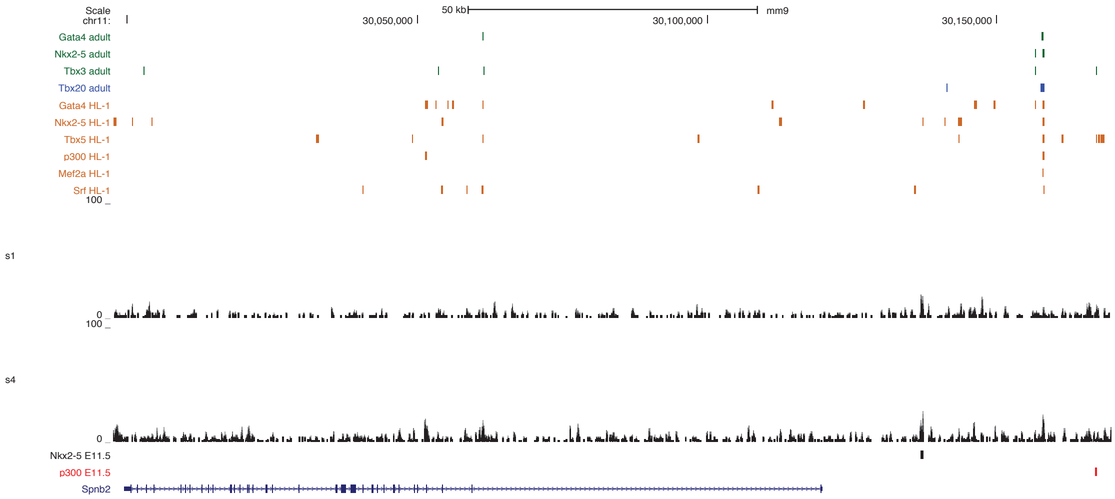

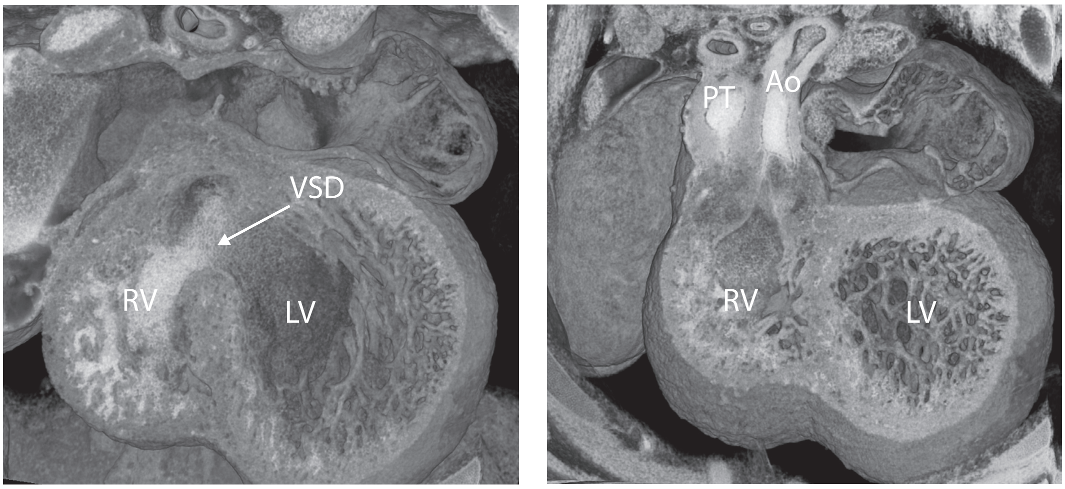

Data S2.
