# Supplementary material for: Sequential Binding of MEIS1 and NKX2-5 on the Popdc2 Gene: A Mechanism for Spatiotemporal Regulation of Enhancers during Cardiogenesis
Source: Cell Rep. 2015 Sep 24;13(1):183–95. doi: 10.1016/j.celrep.2015.08.065 (PMC4597108; doi:10.1016/j.celrep.2015.08.065)
Supplement: Data S2. Description of the Locus and Phenotype Associated with the Six Gene Targets of NKX2-5 Present in the Mouse Phenotype Database, Related to Figure 1 [file mmc3.pdf]

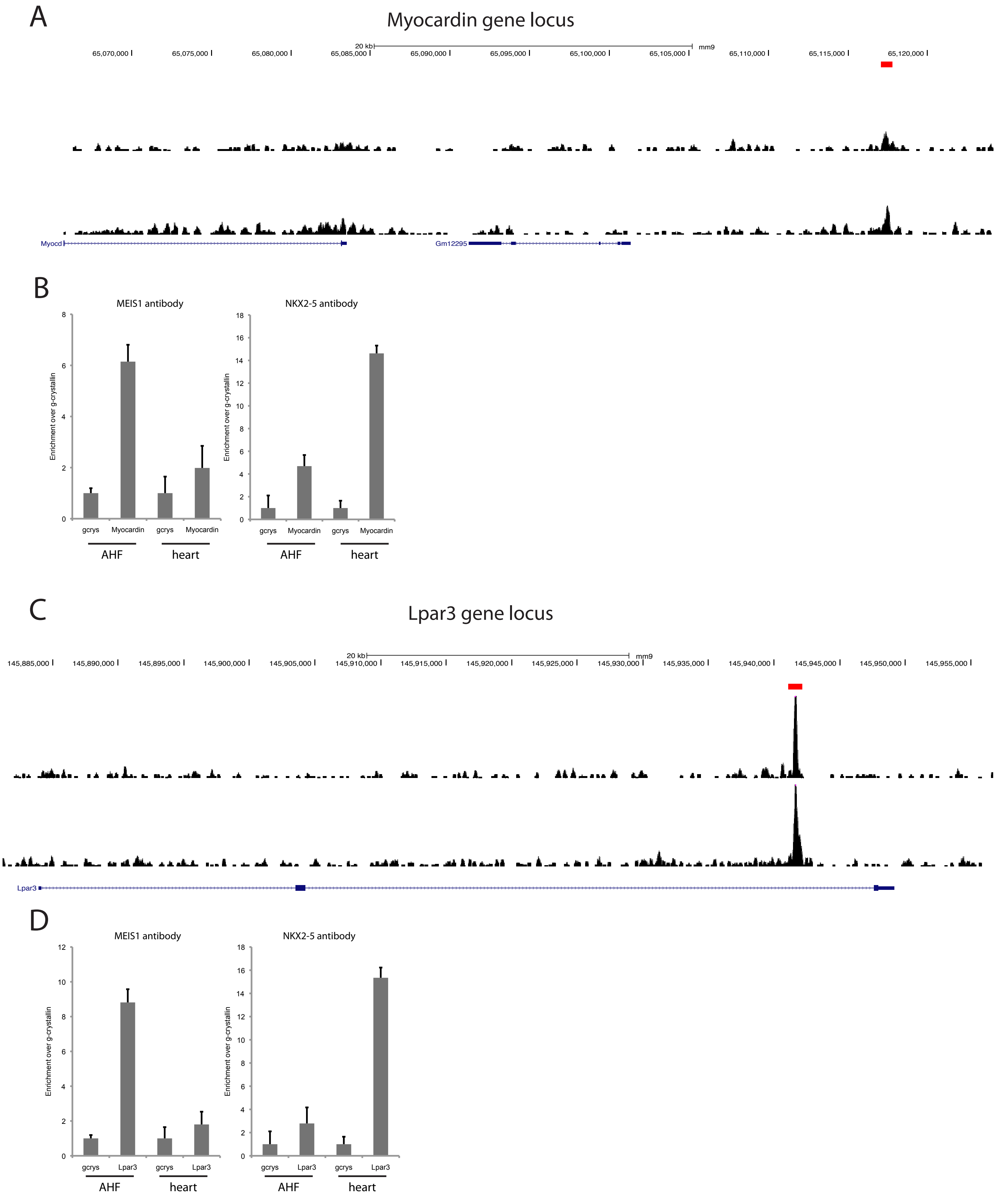

Data S3. MEIS1 and NKX2-5 successively bind four identified enhancers, Related to Figure 4. (A, C, E, F) ChIP-seq trace at the four loci studied. Red bar indicates the region found enriched for NKX2-5 binding and tested in the ChIP assay. (A) Locus of the Myocardin gene. (C) Locus of the Lpar3 gene. (E) Locus of the Gja1 and Hsf2 genes (G) Locus for the Cadm1 gene. (B, D, F, H) ChIP analysis carried out with chromatin purified from E9.5 AHF and heart with either MEIS1 or NKX2-5 antibodies. Representative results of quantitative PCR analysis with primers against the region enriched for NKX2-5 binding are shown. Relative enrichment is presented over a negative control region in the gamma-crystallin gene (gcrys). Two independant experiments were carried out.

E

Gja1 and Hsf2 gene locus

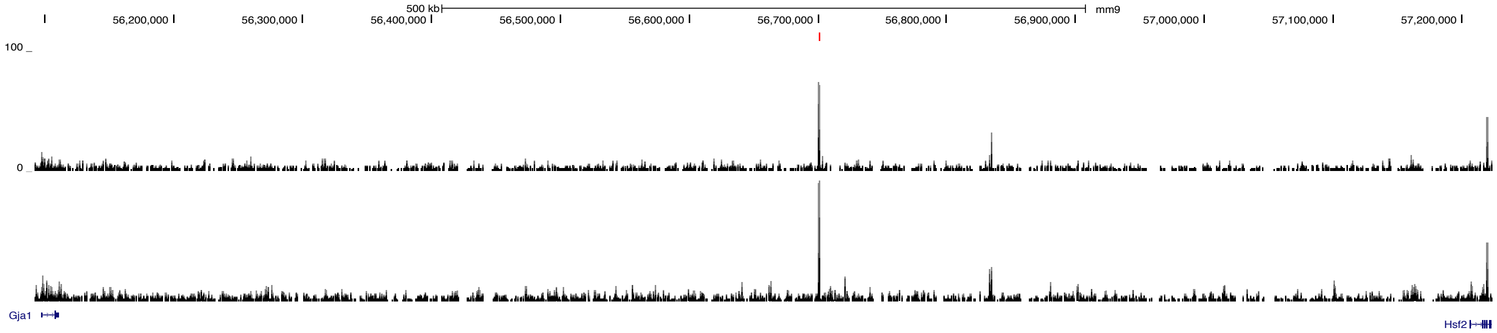

F

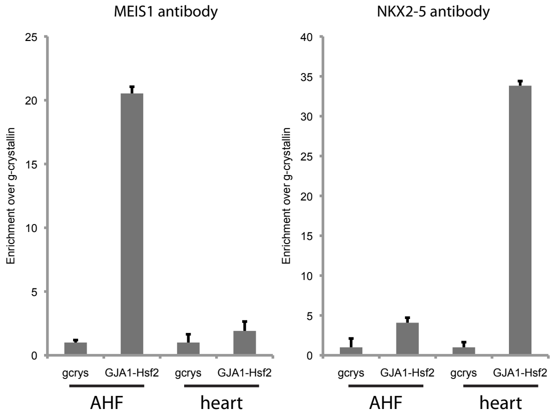

G

Cadm1 genes locus

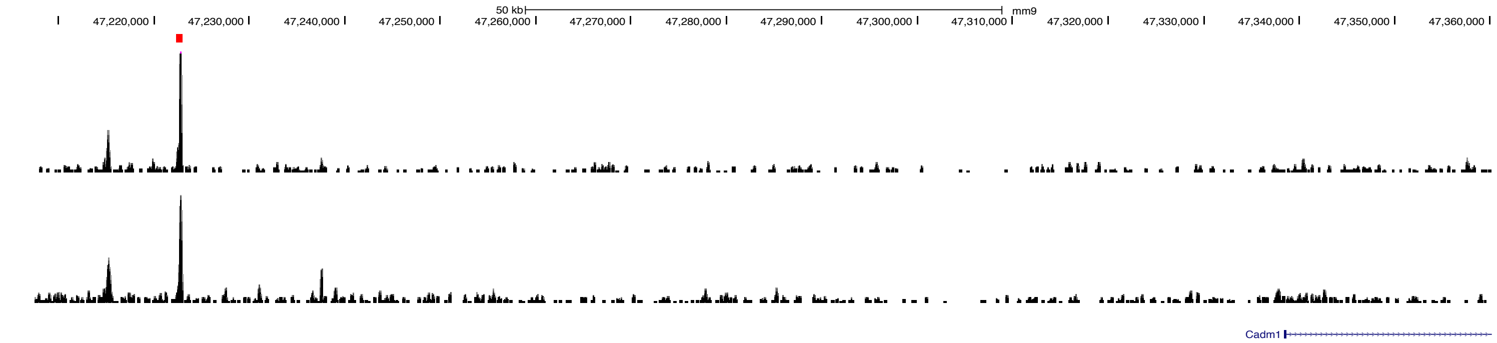

H

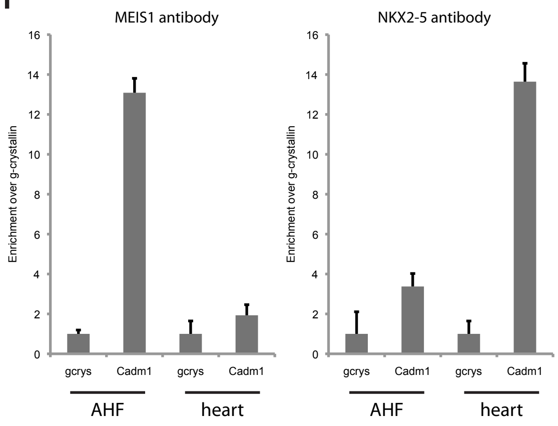

Data S3.
