# Supplementary material for: Sequential Binding of MEIS1 and NKX2-5 on the Popdc2 Gene: A Mechanism for Spatiotemporal Regulation of Enhancers during Cardiogenesis
Source: Cell Rep. 2015 Sep 24;13(1):183–95. doi: 10.1016/j.celrep.2015.08.065 (PMC4597108; doi:10.1016/j.celrep.2015.08.065)
Supplement: Document S2. Article plus Supplemental Information [file mmc6.pdf]

Cell Reports

Article

Sequential Binding of MEIS1 and NKX2-5 on the Popdc2 Gene: A Mechanism for Spatiotemporal Regulation of Enhancers during Cardiogenesis

Graphical Abstract

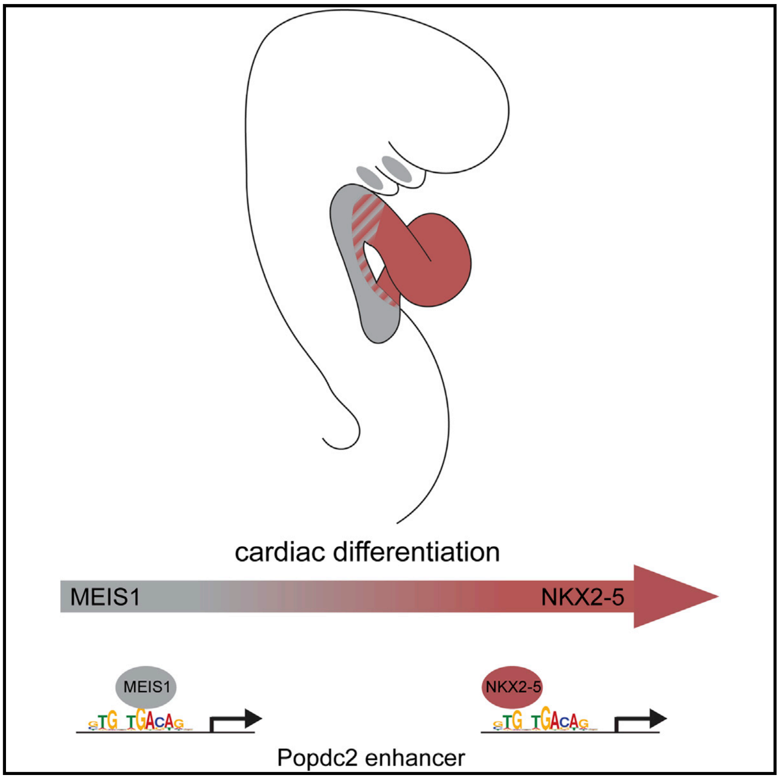

Authors

Laurent Dupays, Catherine Shang, Robert Wilson, ..., Sophie Wood, Norma Towers, Timothy Mohun

Correspondence

tim.mohun@crick.ac.uk

In Brief

Dupays et al. use a combination of ChIP-seq and RNA-seq to identify the direct targets of the homeodomain transcription factor NKX2-5 during cardiac development. They propose a mechanism of transcriptional regulation during cardiac differentiation by successive binding of the two homeodomain transcription factors MEIS1 and NKX2-5.

Highlights

- NKX2-5 shares a DNA-binding site with MEIS1
- MEIS1 and NKX2-5 successively bind a *Popdc2* enhancer
- Successive binding by MEIS1 and NKX2-5 is a general mechanism of regulation
- NKX2-5 represses fast troponin isoforms in the atria

Accession Numbers

GSE44576

Dupays et al., 2015, Cell Reports *13*, 183–195
October 6, 2015 ©2015 The Authors
http://dx.doi.org/10.1016/j.celrep.2015.08.065

Cell Reports
Article

OPEN
ACCESS
CellPress

Sequential Binding of MEIS1 and NKX2-5 on the Popdc2 Gene: A Mechanism for Spatiotemporal Regulation of Enhancers during Cardiogenesis

Laurent Dupays,[1] Catherine Shang,[1] Robert Wilson,[1] Surendra Kotecha,[1] Sophie Wood,[2] Norma Towers,[1] and Timothy Mohun[1,*]
[1]The Francis Crick Institute, Mill Hill Laboratory, the Ridgeway, Mill Hill, London NW7 1AA, UK
[2]Procedural Services Section, The Francis Crick Institute, Mill Hill Laboratory, the Ridgeway, Mill Hill, London NW7 1AA, UK
*Correspondence: tim.mohun@crick.ac.uk
http://dx.doi.org/10.1016/j.celrep.2015.08.065
This is an open access article under the CC BY license (http://creativecommons.org/licenses/by/4.0/).

SUMMARY

The homeobox transcription factors NKX2-5 and MEIS1 are essential for vertebrate heart development and normal physiology of the adult heart. We show that, during cardiac differentiation, the two transcription factors have partially overlapping expression patterns, with the result that as cardiac progenitors from the anterior heart field differentiate and migrate into the cardiac outflow tract, they sequentially experience high levels of MEIS1 and then increasing levels of NKX2-5. Using the *Popdc2* gene as an example, we also show that a significant proportion of target genes for NKX2-5 contain a binding motif recognized by NKX2-5, which overlaps with a binding site for MEIS1. Binding of the two factors to such overlapping sites is mutually exclusive, and this provides a simple regulatory mechanism for spatial and temporal synchronization of a common pool of targets between NKX2-5 and MEIS1.

INTRODUCTION

Congenital heart disease affects up to 1% of all live births in the west and constitutes a major public health burden (Hoffman and Kaplan, 2002). Numerous transcription factors have been shown to play a decisive role in vertebrate heart formation (reviewed in Bruneau, 2008). Identifying the targets of these factors and their regulatory interactions will be a major step toward understanding the broader cardiac developmental program. NKX2-5 is one of the earliest transcription factors expressed in the cardiac lineage, and genetic studies have demonstrated that, from flies to man, the protein has an essential function in heart development (Biben et al., 2000; Tanaka et al., 1999). For example, NKX2-5 has been shown to regulate the size and proliferation of cardiac precursor populations, terminal differentiation of the myocardium, establishment of the ventricular conduction system, and postnatal conduction function (Briggs et al., 2008; Jay et al., 2004; Prall et al., 2007). In humans, mutations in the *NKX2-5* gene cause congenital heart disease; haploinsufficiency results in a spectrum of congenital heart disease of varying phenotypic penetrance, which is mirrored in mouse models (reviewed in Akazawa and Komuro, 2005).

Genome-wide occupancy profiling using chromatin immunoprecipitation (ChIP) recently has been used to identify NKX2-5-binding sites in both cultured cell lines and adult heart (He et al., 2011; van den Boogaard et al., 2012); but, despite the evident importance of NKX2-5 in heart development, no comparable studies have addressed its place within the gene regulatory network that drives cardiogenesis.

Here, we have combined genome-wide ChIP followed by massively parallel DNA sequencing (ChIP-seq) and whole-transcriptome RNA sequencing (RNA-seq) using embryonic hearts from a mouse line that shows hypomorphic expression of NKX2-5 and models human cardiomyopathy. This has enabled us to identify direct targets of the NKX2-5 transcription factor during heart development in vivo. We have found that a large proportion of cardiac enhancers binding NKX2-5 have an overlapping binding site for the homeobox transcription factor, MEIS1, indicating a regulatory interaction between these two proteins. Our data suggest that the combination of shared transcriptional targets, differential DNA-binding affinities, and overlapping expression domains provides a mechanism for spatial and temporal synchronization of a common pool of targets between NKX2-5 and MEIS1.

RESULTS

ChIP-Seq Analysis in the Embryonic Heart Identifies Putative Direct Targets of NKX2-5 with a Critical Role in Heart Structure and Function

We performed ChIP with embryonic day (E)11.5 mouse embryo hearts using an NKX2-5 antibody followed by massive, parallel sequencing (ChIP-seq) to identify 2,610 regions enriched for that factor (Data S1). Of these, 28 were randomly selected for individual validation by ChIP-qPCR, all showing consistent enrichment for NKX2-5 binding in vivo (Figure S1).

Bioinformatic analysis identified 3,313 genes flanking the NKX2-5-binding loci (the two nearest genes for each region enriched in a maximum distance of 1 Mb were considered), and we performed a computational analysis for enrichment of gene ontology (GO) terms associated with those genes. This identified

CrossMark

OPEN
ACCESS
CellPress

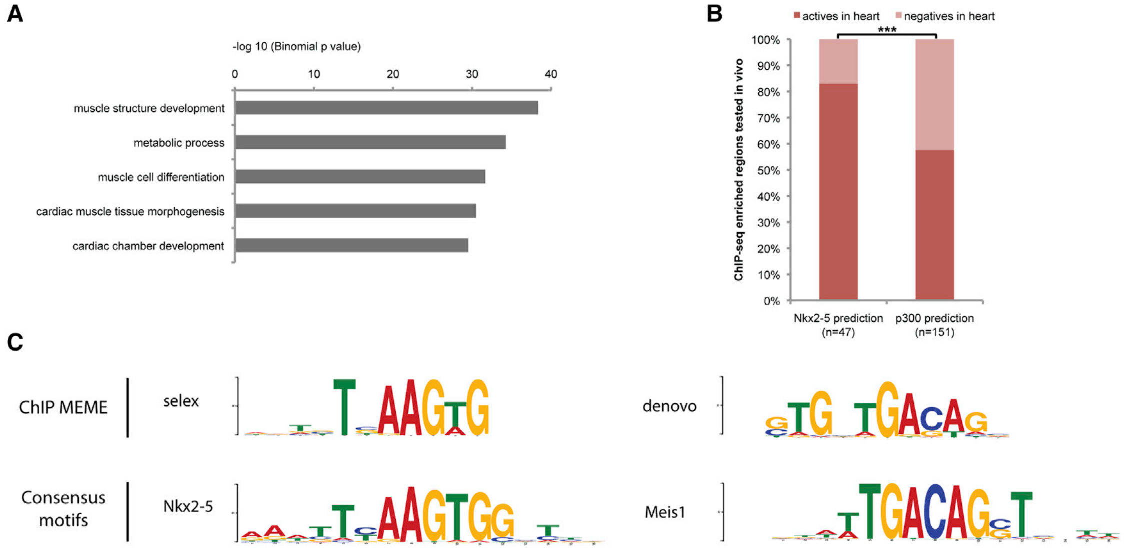

Figure 1. ChIP-Seq Dataset Analysis

(A) Top selected GO terms for biological processes associated with the 3,313 genes identified are shown.

(B) Regions enriched for either NKX2-5 or P300 that have been tested in vivo are plotted according to their ability to drive cardiac expression. Actives or negatives in heart represent the percentage of enhancers able or not able to drive cardiac expression of the β-galactosidase reporter gene ($p < 0.0007$ according to two-tailed Fisher's exact test).

(C) De novo motif discovery using ChIP-MEME identified two in vivo NKX2-5-binding motifs (called selex and de novo). Selex and de novo match the UniProbe consensus motif for NKX2-5 and MEIS1, respectively. See also Figure S3.

significant enrichment of biological process related to muscle structure development and cardiac chamber development (Figure 1A), in agreement with the known role of NKX2-5 as a cardiac transcription factor.

To assess whether the NKX2-5-binding loci are likely to identify transcriptional enhancer regions, we compared them with those enriched for binding of P300 in the heart at the same stage (Blow et al., 2010). P300 is a known marker of enhancer regions and its binding shows a significant overlap with the NKX2-5 ChIP-seq loci (78.4-fold enrichment compared to random regions; see Table S1). Furthermore, by comparing both NKX2-5 and P300 datasets with regions tested for enhancer activity in transgenic mice (as compiled in the Vista enhancer database [Visel et al., 2007]), we found that binding of NKX2-5 was even more reliable for predicting cardiac enhancer activity in the E11.5 mouse heart than binding of P300 (83% versus 58%, respectively; Figure 1B). Our data also show significant overlap with recent studies of cardiac transcription factor binding that have used adult heart or cardiac HL-1 cells (Blow et al., 2010; He et al., 2011; Shen et al., 2011; van den Boogaard et al., 2012; Table S1).

Only a small proportion of putative targets have been the subject of published studies using gene ablation that could reveal cardiac defects. However, several already have been constitutively targeted as part of the EUCOMM/KOMP initiative (Bradley et al., 2012) and subject to comprehensive imaging at E14.5 (http://dmdd.org.uk; Mohun and Weninger, 2011). Six of the eight genes studied in this way and present in our list of targets show clear cardiac developmental defects, consistent with a role in cardiogenesis. All show large ventricular septal defects associated with aberrant connection of the great vessels to the heart chambers (either overriding aorta [OA] or double outlet right ventricle [DORV]). Several show abnormally thin ventricular walls coupled with an increase in the ventricular trabecular network (Data S2). Furthermore, selected NKX2-5-binding sites conferred cardiac expression in a transgenic reporter assay with zebrafish embryos (Figure S2), confirming their ability to act as cardiac enhancers. Taken together with the bioinformatics data, these results indicate that a large proportion of the NKX2-5-binding regions in the embryonic heart that we have identified are likely to be functionally relevant and associated with cardiac development or function in vivo.

Identification of an Overlapping Binding Site Shared by NKX2-5 and MEIS1

Sequence analysis of the 25 bp flanking the summit of each NKX2-5-binding region was undertaken, using a de novo motif-binding program to identify conserved DNA motifs. As expected, the most common of these was a consensus NKX2-5-binding site (hereafter termed "selex"; Figure 1C, e value = 1.6e−24), which matched that previously established for the protein (NKX2-5_3436.1, uniprobe, UP00249, p = 4.71375e−6) as well as the NKX2-5-binding motifs identified in recent ChIP-seq studies with adult heart and HL-1 cells (He et al., 2011; van den Boogaard et al., 2012; Figure S3).

Surprisingly, the de novo motif-binding program also identified a second common consensus (hereafter termed "de novo"; Figure 1C, e value = 6.5e−95) that appears to be a hybrid site: its 3′ portion matches a binding motif previously identified for the homeodomain protein MEIS1 (MEIS1_2335.1, uniprobe,

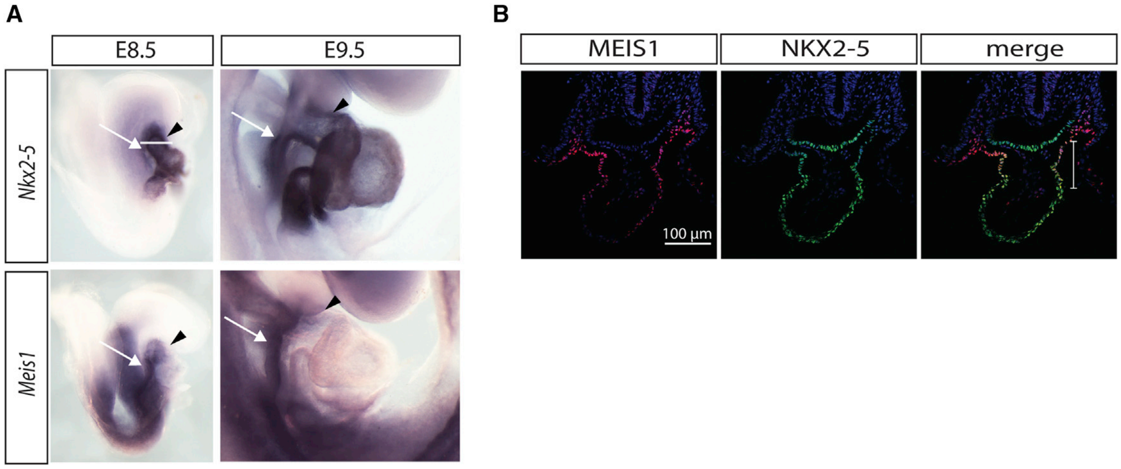

Figure 2. NKX2-5 and MEIS1 Expression Pattern

(A) *Nkx2-5* and *Meis1* whole-mount in situ hybridization in E8.5 and E9.5 mouse embryos shows their co-expression in the anterior heart field (AHF, white arrows) and outflow tract (OFT, arrowheads).

(B) NKX2-5 and MEIS1 immunohistochemistry on an E8.5 mouse embryo (transversal section) showing colocalization in the distal OFT (bracket in merge). Level of section is shown with a white line in (A). AHF and heart tube (HT) are depicted in the MEIS1 immunostaining.

UP00186, p = 0.000185), while the 5′ is similar to the selex (NKX2-5)-binding motif. The hybrid, de novo motif, therefore, appears to comprise an overlapping binding site for the two transcription factors. Using the Find Individual Motif Occurrences (FIMO) software package to analyze regions immediately encompassing the summit of NKX2-5 binding (±25 bp), we found that the de novo motif is present in 53.4% of binding regions, compared with 42% for the selex motif. Only 4.6% of the regions analyzed contained both the de novo and selex consensus sequences. These data suggest that, in vivo, a significant proportion of NKX2-5 binding on cardiac enhancers occurs via an overlapping site shared with MEIS1.

To test the possible biological relevance of a binding site shared by NKX2-5 and MEIS1, we first re-assessed the relative expression patterns of the two transcription factors in the early embryo. At E8.5 *Nkx2-5* is strongly expressed in the cardiac tube, with weaker expression in the adjacent anterior heart field (AHF) (Figure 2A, black arrowhead and white arrow, respectively). At the same stage, although *Meis1* expression is undetectable in much of the heart tube, it is strongly expressed in both the AHF and in the distal part of the outflow tract (OFT), overlapping at this stage with the expression domain of *Nkx2-5*. At E9.5, expression of both *Nkx2-5* and *Meis1* persists strongly in the AHF while levels of *Meis1* in the distal OFT are reduced (Figure 2A). Coexpression of MEIS1 and NKX2-5 within the distal OFT of the heart tube at E8.5 was confirmed by immunohistochemistry (Figure 2B).

Using electrophoretic mobility shift assays (EMSAs), we confirmed that both proteins can indeed bind in vitro to the de novo motif identified from ChIP-seq (Figure 3A; Figure S3B). By comparing the effectiveness of the de novo and selex sequences to compete for binding, we established that NKX2-5 has a much higher affinity for the selex sequence compared with the de novo motif (Figure 3B). Moreover, when tested together, we could find no evidence for simultaneous binding of both proteins to the de novo sequence, rather each apparently bound independently (Figure 3C, arrow and arrowhead). Titration experiments demonstrated that even increasing the amount of either protein failed to produce an additional larger-sized complex (Figure 3C). Indeed, at higher concentrations of MEIS1 protein, NKX2-5 binding disappeared, (Figure 3C, compare lane 3 to lanes 10 and 11, red arrows). Interestingly, at higher NKX2-5 doses, although MEIS1 binding was inhibited, there was not the expected further increase in NKX2-5 binding. One possible explanation for this behavior is that, at higher concentrations, NKX2-5 may sequester MEIS1 in a direct complex that can bind neither site.

Our EMSA results indicate that binding of NKX2-5 and MEIS1 on the de novo motif is mutually exclusive. Consistent with this, in a luciferase assay, individually both NKX2-5 and MEIS1 were able to transactivate reporter expression via the de novo sequence, but we found no evidence of synergistic activation by the two factors (Figure 3D).

MEIS1 and NKX2-5 Successively Bind to a Popdc2 Enhancer

The Popdc2 gene (1 kb; Figure 4A, black bar) acts as an enhancer, driving cardiac expression of a fluorescent reporter in transgenic fish (Figure S2). A de novo DNA-binding site was found in the region enriched for NKX2-5 binding, 110 bp upstream of the Popdc2 transcription start site (Figure 4A). The same region also was found to be enriched for MEIS binding within the first branchial arch of the early embryo (Amin et al., 2015 and see below). This sequence also drives cardiac expression in the developing mouse heart (Figure 4B). Analysis of representative transient transgenic embryos at E9.5 showed that the enhancer-driven transgene recapitulated normal expression in differentiated cardiomyocytes of the cardiac tube and cardiac precursors lying in the AHF (Froese and Brand, 2008). Expression of the transgene also was observed in the pharyngeal

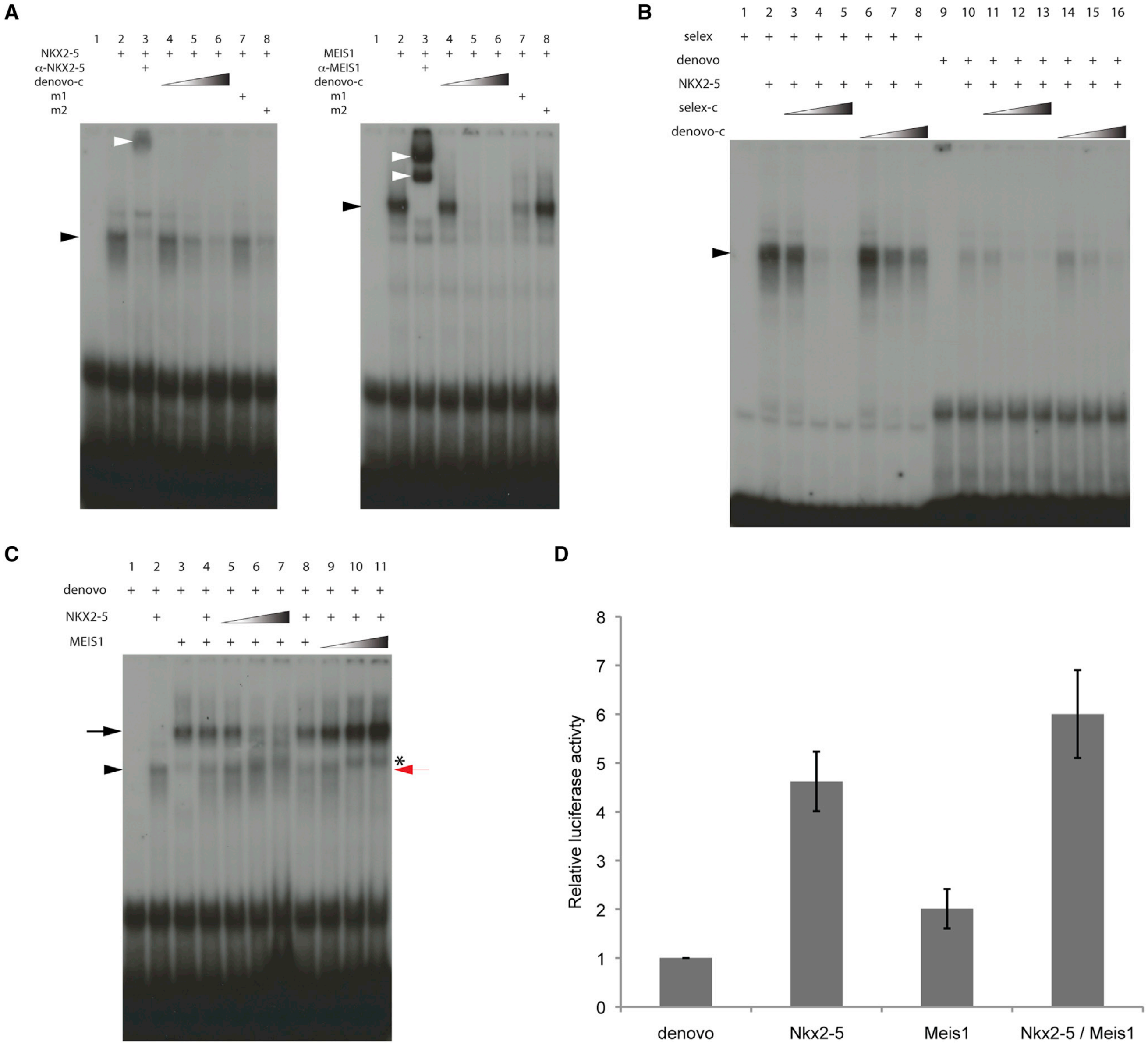

Figure 3. NKX2-5 and MEIS1 Bind on the Same DNA Motif

(A) Electrophoretic mobility shift assay (EMSA) showing that NKX2-5 and MEIS1 bind the de novo probe (black arrowheads). Note the specific competition when an unlabelled competitor (de novo-c) is used. White arrowheads indicate supershift when a specific antibody against the protein tested is used. Note that competition with a mutated unlabelled probe is observed with de novo m2, but not de novo m1, when NKX2-5 protein is used. Competition with a mutated unlabelled probe is observed with de novo m1, but not m2, when MEIS1 protein is used.

(B) The selex unlabelled probe is a better competitor than the de novo unlabelled probe when used with selex or de novo radioactive probes. Black arrowhead indicates NKX2-5 probe complex.

(C) EMSA showing the absence of any supershift when both NKX2-5 and MEIS1 proteins are added in increasing amounts. Increasingly prominent band slightly above the position of the MEIS1 complex in lanes 10 and 11 (indicated with *) is detectable in the presence of MEIS1 alone in lane 3, and its apparent increase simply reflects the increase in overall MEIS1 concentration in the titration experiment. Black arrow and black arrowhead indicate MEIS1 and NKX2-5 binding on the de novo probe, respectively. Note the disappearance of NKX2-5 binding in lanes 10 and 11 at higher concentrations of MEIS1 (red arrow).

(D) NKX2-5 and MEIS1 activate the de novo fragment cloned in pGL3 in 3T3 cells. Note the absence of synergistic activation. The graph shows relative luciferase reporter activity normalized to reporter construct alone. Average of three independent experiments is shown. Error bars represent the SEM.

arch and the proepicardium (Figure 4B, asterisk and white arrow), as observed for the *Popdc2* gene itself (Froese and Brand, 2008). *Meis1* expression also was observed in these tissues, but no expression of *Nkx2-5* could be detected (data not shown). Moreover, *Popdc2* RNA expression was downregulated in Nkx2-5-null hearts at E9.0 compared with controls (Figure S4A).

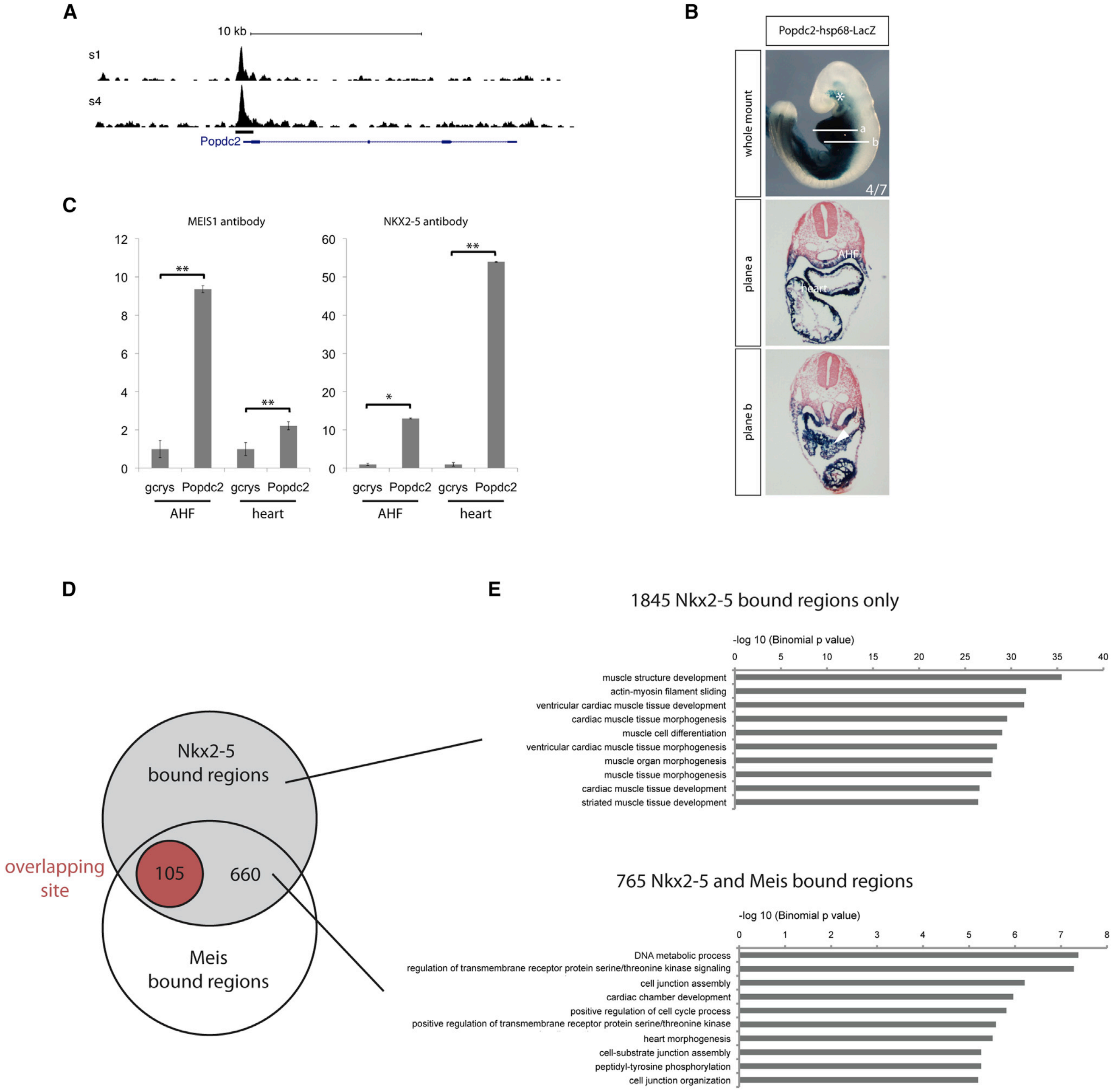

Figure 4. NKX2-5 and MEIS1 Successively Bind a Popdc2 Enhancer

(A) ChIP-seq trace at the Popdc2 locus for the two experiments carried out (s1 and s4). Black bar indicates the region found enriched for NKX2-5 binding and tested in transgenic mice (see below).

(B) Representative image of a mouse transgenic embryo at E9.5 showing that the Popdc2 enhancer is reproducing endogenous Popdc2 gene expression in the heart and AHF. Four independent embryos expressed the reporter gene of the seven PCR-positive embryos for the transgene. Planes a and b represent transversal sections as indicated on the whole mount. Asterisk shows pharyngeal arches, white arrow in plane b shows proepicardium, and white arrowhead indicates expression of the transgene in the AHF.

(C) ChIP analysis carried out with chromatin purified from E9.5 AHF and heart with either MEIS1 or NKX2-5 antibodies. Representative results of qPCR analysis with primers against the region of the Popdc2 promoter enriched for NKX2-5 binding are shown. Relative enrichment is presented over a negative control region in the gamma-crystallin gene (gcrys). $*p < 0.05$, $**p < 0.001$ according to a two-tailed Student's t test.

(D) Venn diagram depicting the overlap between NKX2-5- and MEIS-enriched regions. In red are the number of those overlapping regions corresponding to the overlapping binding site.

(E) Top selected GO terms were identified with GREAT for biological processes associated with the genes associated with either the NKX2-5- or NKX2-5/MEIS-bound regions.

A similar, though less profound effect was observed at E11.5 in hearts that are hypomorphic for NKX2-5 expression (Figure S4B). These data indicate that NKX2-5 plays a major role in regulating the expression of *Popdc2*.

To examine directly the relative binding of MEIS1 and NKX2-5 in vivo, we performed ChIP experiments on the Popdc2 enhancer using chromatin from samples prepared from the AHF and heart of E9.5 embryos. NKX2-5 binding on the Popdc2 enhancer was found to be much stronger in chromatin extracted from the embryonic heart compared to the adjacent AHF (Figure 4C). Conversely, MEIS1 bound more strongly in chromatin extracted from the AHF rather than the heart. The relatively low level of enrichment found for MEIS1 in the heart and NKX2-5 in the AHF parallels the low level of expression of these factors within the respective tissues (Figure 2). These results indicate that MEIS1 binding on the *Popdc2* enhancer predominates in the AHF, while NKX2-5 binding predominates in the heart.

To assess whether other gene enhancers containing the de novo DNA-binding motif showed similar tissue-specific differential binding, we tested four such genomic enhancer regions identified by ChIP-seq for Nkx2-5 in the embryonic heart (Myocardin, Lpar3, Cadm1, and Gja1/Hsf2 loci; Data S3). All showed a pattern of enrichment for MEIS binding in the AHF and NKX2-5 binding in the heart, suggesting that this behavior is likely to be shared by many of the genomic regions harboring the de novo site identified by our ChIP-seq study.

MEIS1 and NKX2-5 Bind to the Same Regions In Vivo

It is well established that the mesodermal core of the first and second branchial arches contains cardiac progenitor cells, which migrate from this region to populate the OFT and chambers of the developing heart (Lescroart et al., 2010). A recent study has documented MEIS genomic occupancy in the branchial arches (Amin et al., 2015), enabling us to compare on a genome-wide basis the loci binding MEIS in the AHF with those binding Nkx2-5 in the heart.

Strikingly, 765 of the 2,610 regions enriched for NKX2-5 binding (29.31%) also were identified by MEIS binding in the first branchial arches. A similar overlap was evident with the second branchial arches (787 regions or 30.15%; Table S1; Figure 4D). This represents a 50-fold enrichment compared to random (Table S1), lending further support to the suggestion that the successive binding of MEIS and NKX2-5 that we initially observed for *Popdc2* is shared by an entire subset of cardiac enhancers.

In an attempt to discriminate between the occurrence of closely associated and directly overlapping binding sites for MEIS1 and NKX2-5, we repeated this analysis but restricted the comparison to a region of ±6 bp around the summit of the binding peaks identified in the different datasets. Under these conditions, 105 binding sites overlapped between NKX2-5 and MEIS in the first branchial arch and, therefore, most likely represent direct overlap of the two binding sites. Comparable analysis yielded 79 common, overlapping sites in the second branchial arch. These results suggest that a significant fraction of MEIS and NKX2-5 interaction in vivo is mediated by a direct site overlap.

Interestingly, GO analysis suggests that the subset of genes regulated by both MEIS1 and NKX2-5 may have a distinct function. The 1,845 regions that bound NKX2-5 alone are associated with genes involved with cardiac muscle structure and development; in contrast, the 765 regions that bound both MEIS1 and NKX2-5 are primarily associated with genes involved with DNA metabolic processes, signaling, and cell-cycle regulation (Figure 4E). No significant difference was evident in GO terms when we divided the 765 between those showing the closest association of MEIS and NKX2-5 binding (105) and the rest (660).

Identification of Putative Direct Targets for NKX2-5 Reveals a Dual Role as Both Activator and Repressor

Comparison of ChIP-seq and transcriptomic data provides a powerful way to identify likely transcriptional targets by identifying those that both bind the transcription factor and are dysregulated in its absence (Sakabe et al., 2012). For NKX2-5, this strategy is precluded since *Nkx2-5* knockout embryos die early (E9.5) due to failure of heart development (Biben et al., 2000; Tanaka et al., 1999). As an alternative, we took advantage of a previously generated mouse model showing hypomorphic expression of NKX2-5 (Prall et al., 2007; Stanley et al., 2002), enabling us to test the effect of reduction, rather than absence, of NKX2-5 expression. Mendelian analysis showed no change in mortality of this hypomorphic line at E11.5, E12.5, and E13.5. However, by E14.5, only 60% of the expected number of hypomorphic embryos were recovered (Table S2) and only a few hypomorphic embryos were obtained at birth (none surviving past weaning). Consistent with these observations, the structure of the hypomorph heart showed profound abnormalities by E14.5, including thinning of ventricular walls, the presence of large ventricular septal defects, and signs of hypertrabeculation (Figure 5A).

In contrast, at E11.5, the hearts of hypomorphic embryos were morphologically indistinguishable from those of sibling controls, and we therefore used this stage for transcriptome analysis. RNA-seq comparing E11.5 hypomorphic mutant and control hearts identified statistically significant alterations in the expression of 1,492 genes (Data S4). By using this to filter the ChIP-seq data, we were able to identify 309 genes as high-probability, direct targets of NKX2-5 (Figure 5B). GO term analysis of these indicated that the categories of biological process most enriched are related to cell signaling and differentiation (Figure 5C, $p < 0.002$ for all categories). Furthermore, the most enriched biological pathways are related to signaling (Figure S4D). Interestingly, of the 309 identified genes, 172 (55.6%) were downregulated and 137 (44.4%) were upregulated, suggesting that NKX2-5 functions both as a transcriptional activator and as a repressor (Figure 5B).

One role of NKX2-5 suggested by studies of the null mutant is in the regulation of cardiac chamber development (Dupays et al., 2005; Tanaka et al., 1999). We tested this hypothesis by classifying the direct NKX2-5 targets identified in our studies according to their expression pattern in the hypomorphic heart, taking advantage of previous studies that examined genes differentially expressed between chamber and non-chamber myocardium at E10.5 (Horsthuis et al., 2009). This enabled us to distinguish between genes restricted to the atrioventricular canal (AVC) or working myocardium, compared with those more broadly

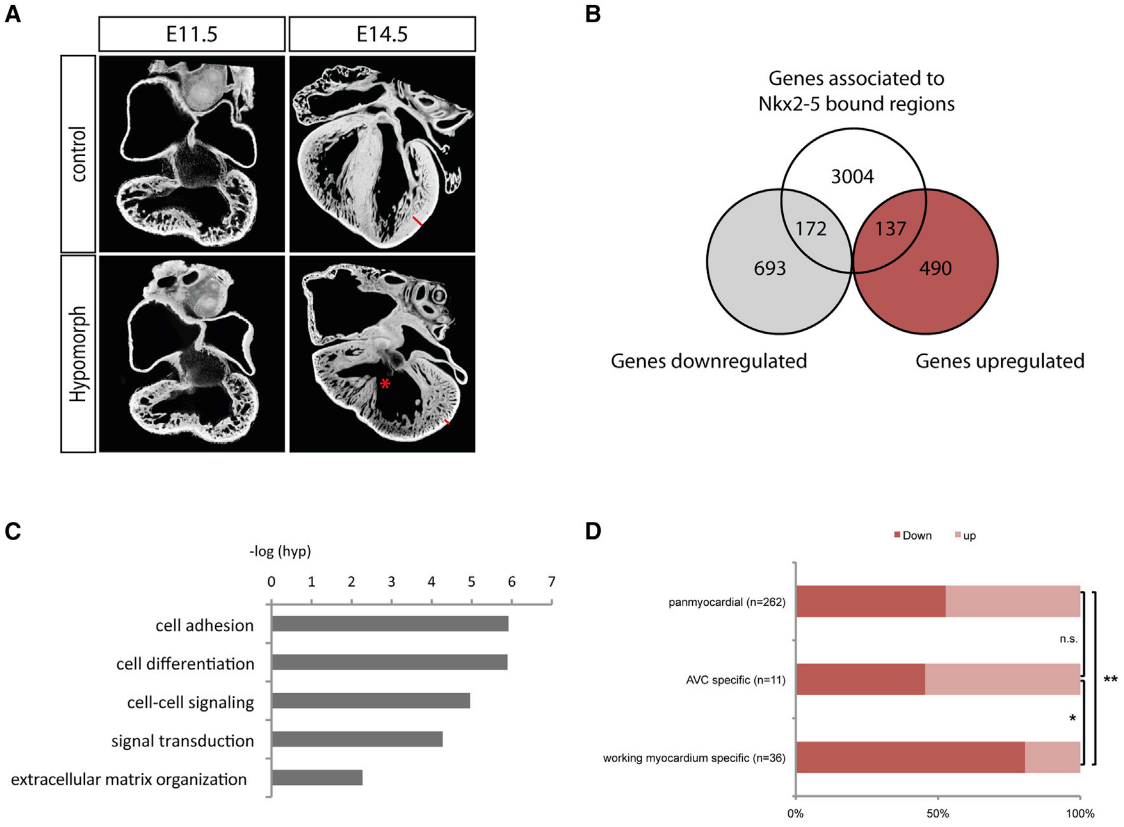

Figure 5. NKX2-5 Acts as an Activator and a Repressor

(A) Four-chambered view comparing hearts from hypomorphic embryos to controls using high-resolution episcopic microscopy. At E11.5, no obvious morphological defect can be detected. At E14.5, hearts from hypomorphic embryos present ventricular septal defect (star) and thinning of the compact layer ventricular free wall (compare red size bar).

(B) Venn diagram depicts the overlap between NKX2-5-enriched region-associated genes (white) and genes downregulated (gray) and upregulated (red) in the heart of hypomorphic embryo.

(C) Most enriched biological processes in the direct target genes list are shown.

(D) Direct targets are categorized by expression pattern as AVC specific or working myocardium. n.s., non-significant; **$p < 0.002$, *$p < 0.0489$ according to two-tailed Fisher's exact test. See also Figure S4D.

expressed (and classified as "pan-myocardial"). Those expressed in the AVC or throughout the myocardium were upregulated or downregulated in the hypomorph in approximately equal proportions (Figure 5D). In contrast, those NKX2-5 targets whose expression was restricted to the chamber myocardium were predominantly downregulated in the mutant (Figure 5D), consistent with an essential role for NKX2-5 in promoting cardiac chamber formation.

NKX2-5 Directly Represses the Expression of Tnnt3 and Tnni2 in the Atria

Our finding that almost half of the putative direct targets of NKX2-5 identified by intersection of ChIP-seq and transcriptomic data are upregulated in the hypomorph is striking, since most previous studies have focused on the role of NKX2-5 as a transcriptional activator (Tanaka et al., 1999). One noteworthy example of negative regulation revealed by our study is associated with binding of NKX2-5 to a region 1 kb upstream of the Lsp1 gene transcription start site (Figure 6A) harboring a selex DNA-binding site (referred as Tnni2-Tnnt3-enh). Lsp1 showed no detectable expression in cardiomyocytes at E11.5, its expression being restricted to a population of cardiac fibroblasts and remaining unchanged in the hypomorph at E14.5 (Figure 6B). However, the two skeletal troponin T genes, Tnni2 and Tnnt3, are located 38 kb upstream and 17 kb downstream, respectively, of the Lsp1-associated NKX2-5-binding region. Furthermore, reduced expression of NKX2-5 expression in the hypomorph heart resulted in upregulation of both troponin genes in the myocardium at E11.5 and E14.5 (Figure S5A; Figure 6B). That the expression of the two skeletal troponin isoforms was restricted to the atrial myocardium of the developing heart indicates further levels of control that distinguish atrial from ventricular myocardium.

We investigated the putative repressor function of NKX2-5 using short hairpin RNA technology to knock down NKX2-5 expression in the HL-1 cardiac cell line. Cells stably transfected with *shNkx2-5* showed a significant decrease in NKX2-5 protein expression (Figure S5B). Using these, we tested the effect of

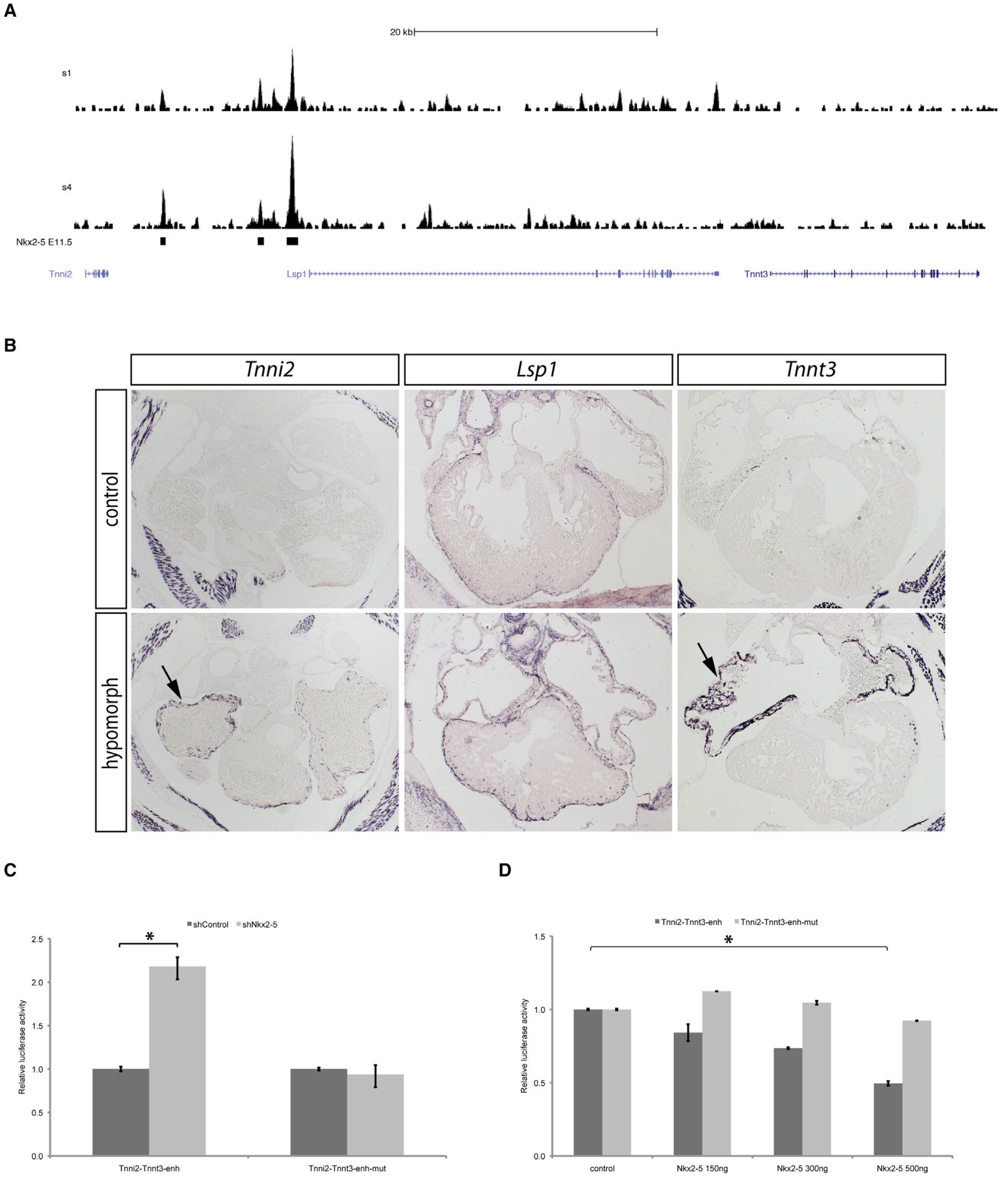

Figure 6. NKX2-5 Represses Cardiac Expression of Fast Troponin Isoforms

(A) ChIP-seq trace at the repressed Lsp1 locus for the two experiments carried out (s1 and s4). Black bars indicate regions found enriched for NKX2-5.

(B) In situ hybridization on sections of E14.5 control and hypomorph hearts, using probes for Tnni2, Tnnt3, and Lsp1. Note the upregulation of Tnni2 and Tnnt3 signals in the hypomorph atria (black arrows). See also Figure S5A.

(legend continued on next page)

NKX2-5 knockdown on the expression of a luciferase reporter carrying the 461-bp Tnni2-Tnnt3-enh fragment (Figure 6C). Luciferase activity was significantly enhanced in HL-1 cells deficient for NKX2-5 compared with controls, but unchanged after mutation of the selex DNA-binding site (Tnni2-Tnnt3-enh-mut; Figure 6C). Furthermore, co-transfection with increasing amounts of an NKX2-5 expression plasmid progressively repressed luciferase reporter activity, but had no effect after mutation of the selex site (Figure 6D). Together, these results demonstrate the ability of NKX2-5 to repress activity from the Tnni2-Tnnt3-enh enhancer sequence.

DISCUSSION

This study describes the genome occupancy of NKX2-5 during a critical phase of cardiac differentiation in the mouse embryo. Our data show that NKX2-5 binding is an efficient predictor of cardiac enhancer location and even more effective than the binding of P300 at the same stage (Blow et al., 2010).

NKX2-5 Can Bind through an Overlapping Site with MEIS1 In Vivo

Our analysis demonstrates that, in vivo, not only is NKX2-5 able to bind to the conventional NKX2-5-binding motif, but it also binds to a related sequence that overlaps with the DNA-binding site for the transcriptional cofactor MEIS1. Comparison of MEIS binding in cardiac precursor tissue of the branchial arches with NKX2-5 in the heart shows that those two factors bind on a common group of enhancers during cardiac differentiation.

A potential role for MEIS proteins as regulators of cardiomyocyte differentiation has been suggested previously (Paige et al., 2012; Wamstad et al., 2012). Consistent with this, in the mouse embryo, ablation of MEIS1 results in prenatal death, the null embryos having severe developmental abnormalities (Azcoitia et al., 2005; Hisa et al., 2004), including ventricular septal defects and an overriding aorta (Stankunas et al., 2008). These are reminiscent of the phenotype found in embryos showing hypomorphic levels of NKX2-5 (Figure 5A; Prall et al., 2007). Recently, MEIS1 also has been shown to regulate postnatal cardiomyocyte proliferation (Mahmoud et al., 2013). Furthermore, genome-wide association studies have linked both *Nkx2-5* and *Meis1* to abnormalities in the PR interval, a region of the electrocardiogram (ECG) that reflects atrial and atrioventricular nodal conduction (Pfeufer et al., 2010; Smith et al., 2011). Given that defects in conduction are associated with mutations in NKX2-5 in both human and mouse models, our finding that the two factors share a common set of gene targets might account for these results.

We have found that, in the early mouse embryo heart, MEIS1 is co-expressed with NKX2-5 protein in the AHF and the OFT. Levels of MEIS1 are higher in the AHF and decrease through the outflow toward the ventricular myocardium. NKX2-5 shows a reciprocal pattern of early expression, with levels in the myocardium being considerably higher than those found in the AHF.

In a recent study, Amin et al. (2015) analyzed the genomic occupancy of MEIS factors in the branchial arches, identifying a very large number (>60,000) of binding sites. They noted that the top 1% of MEIS-binding regions were associated with genes involved in skeletal muscle development (see Figure 5F in Amin et al., 2015). Comparison with the NKX2-5-binding data we have obtained now reveals that a significant fraction of NKX2-5-dependent cardiac enhancers harbors an overlapping MEIS site.

The developmental period during which cells migrate from the AHF into the developing heart coincides with a transition from cardiac progenitors to differentiated cardiomyocytes, resulting in many well-documented changes in gene expression (Domian et al., 2009; Wamstad et al., 2012) and cell cycling (Cai et al., 2005; van den Berg et al., 2009). These coincide with the period over which we identify the switch in expression from MEIS1 to NKX2-5. It is, therefore, tempting to speculate that the subgroup of MEIS1-regulated genes that shows overlapping binding by NKX2-5 is associated with these differentiation changes.

Consistent with this, we found that 79 of the genes associated with those 765 regions are in common with target genes associated with enhancers containing the MEIS1 motif in differentiated cardiac embryonic stem cell (Wamstad et al., 2012). Furthermore, using previously published data that identifies genes enriched in the AHF compared to the heart and vice versa (Domian et al., 2009), we found that 51 of the genes associated with NKX2-5/MEIS-bound regions are AHF enriched, while 76 are from the heart-enriched class (see Figure S4C). Many of these genes have well-described expression patterns and have been associated with changes in DNA metabolism, cell cycling, and cell signaling (e.g., Tbx20, myocardin, Cdh2, Wnt11, and Wnt2), which are consequent upon cardiac progenitor differentiation. Together, these results suggest that enhancers binding both MEIS1 and NKX2-5 constitute a functionally distinct class of genes compared with those regulated by MEIS1 alone.

Given that early heart growth largely results from the addition of cells from the AHF to the poles of the heart (Cai et al., 2003; Kelly, 2012), our data suggest that a significant portion of NKX2-5-binding regions are regulated through successive binding first by MEIS1 and later by NKX2-5 as differentiation (and cardiac morphogenesis) proceeds. Interestingly, a similar temporal pattern of expression of those factors is found during embryonic stem differentiation into beating cardiomyocytes (Wamstad et al., 2012). In such stem cell cultures, cardiomyocyte precursors express high levels of *Meis1* RNA and relative low levels of the *Nkx2-5* transcript. However, when such precursors differentiate into cardiomyocytes, *Meis1* RNA expression decreases and *Nkx2-5* RNA expression increases (see Figure S2 in Wamstad et al., 2012).

Transcriptional regulation in a similar manner through shared binding sites and overlapping but distinct spatial patterns of transcription factor expression already has been described during cardiogenesis, from studies of the growth factor FGF10.

(C) Tnni2-Tnnt3 enhancer (Tnni2-Tnnt3-enh) activity in control and NKX2-5-deficient HL-1 cells is shown.

(D) Co-transfection of Tnni2-Tnnt3-enh with an increasing quantity of *Nkx2-5*-expressing vector shows a decrease of reporter activity. Tnni2-Tnnt3-enh-mut reporter vector is unaffected by *Nkx2-5* overexpression in HL-1 cells. Only significant changes are indicated. *$p < 0.05$ according to a two-tailed Student's t test.

An *Fgf10* enhancer possesses a shared binding site for NKX2-5 and the transcription factor ISL1. *Fgf10* is activated in the AHF by ISL1, but subsequently repressed by NKX2-5 as cells of the AHF are added to the heart tube (Watanabe et al., 2012). Our data now suggest a similar regulatory mechanism with MEIS1 and NKX2-5, and we speculate that the presence of overlapping transcription factor binding sites in gene enhancers might therefore be of broader significance during development. For gene enhancers containing such overlapping binding sites, distinct regional patterns of transcription factor expression could combine with cell migration to achieve complex patterns of spatiotemporal regulation.

Common targeting of a set of genes by MEIS1 and NKX2-5 could facilitate several different regulatory mechanisms underlying cardiac differentiation. For example, preferential binding by MEIS1 within the AHF could act to prevent activation of a set of cardiac differentiation genes normally activated by NKX2-5; these genes subsequently would become activated as AHF cells are added to the cardiac tube and MEIS1 is replaced by NKX2-5. An alternative possibility is a role for MEIS1 in activating genes that are part of a cardiac progenitor state program in the AHF. As cardiac precursors integrate into the developing heart, replacement of MEIS1 by NKX2-5 would serve to repress these genes and, thereby, allow cardiomyocyte differentiation. Consistent with this latter model, Nkx2-5 mutants have been reported to show upregulation of progenitor signature genes in the AHF and abnormal persistence of their expression in differentiating myocytes (Prall et al., 2007). Furthermore, our results show that there is a clear difference between biological processes associated with genes showing NKX2-5 binding and those regulated by both NKX2-5 and MEIS. The former are specific to the differentiated cardiac state, whereas the latter are related to other biological processes (metabolism, signaling, and cell-cycle regulation), which perhaps reflects their role in a cardiac progenitor gene program.

A third possibility is suggested by accumulating evidence that gene enhancers can interact with pioneer transcription factors at early stages of development before the target genes are even transcribed (Kathiriya et al., 2015). For example, in the branchial arches, HOXA2 and MEIS binding largely overlaps and it has been suggested that MEIS binding functions to create an accessible platform recognized by HOXA2 (Amin et al., 2015). Similarly, in the mouse embryo trunk, Hox target sequences have been shown to strongly associate with MEIS factors (Penkov et al., 2013). Perhaps when both MEIS1 and NKX2-5 activate a common set of enhancers, MEIS1 establishes a ground state that maintains competence for eventual activation by NKX2-5 at the appropriate stage of cardiac differentiation.

Genome-wide studies of NKX2-5-binding sites in the adult mouse heart show no statistically significant enrichment for the de novo binding motif (van den Boogaard et al., 2012; data not shown), in contrast to our findings for the E11.5 embryonic heart. This may reflect the exclusive developmental role of the MEIS/NKX2-5 combination, but it also could be accounted for by the nature of ChIP studies themselves. Since no MEIS1 is expressed in the embryonic heart, enhancers containing the de novo binding site will be recovered by ChIP using an NKX2-5 antibody. In contrast, as the adult heart expresses MEIS1, this factor will compete with NKX2-5 for binding onto the de novo motif. Preferential binding by MEIS1 in vivo (comparable with that we detected in vitro) would reduce or abolish recovery of sequences containing the de novo consensus by NKX2-5 ChIP.

NKX2-5 Represses the Muscle Fast Fiber Phenotype

NKX2-5 previously has been shown to be capable of acting as a transcriptional repressor (Prall et al., 2007). Our study provides evidence of how widespread such a role may be during development, with nearly 50% of the putative direct targets identified in the embryonic heart likely to be regulated by NKX2-5 in this manner.

Our study of the NKX2-5 hypomorph embryos revealed an unexpected role for NKX2-5 that is notable in several ways. NKX2-5 apparently regulates skeletal muscle fiber type and does so via repression of two skeletal fast isoforms of troponin. Individual isoforms show quantitative differences in the calcium activation of contraction that can affect cardiac muscle performance and heart contraction (Huang et al., 2008; Parmacek and Solaro, 2004). The strong upregulation of Tnnt3 and Tnni2 that occurs in the developing atria of the NKX2-5 hypomorph is likely, therefore, to result in alteration of contractile performance, through its impact on myofibrillar calcium activation. The morphological abnormalities seen in the hypomorph hearts at E14.5 included changes (wall thinning and atrial distension) indicative of compromised cardiac function. Our findings indicate that this might not simply be an indirect and cumulative consequence of cardiac dysfunction in the hypomorph; it may in fact be largely driven by a direct impact of reduced NKX2-5 levels on atrial myofibrillar composition. Interestingly, a similar upregulation of fast skeletal troponin isoforms has been observed in several models of cardiac failure induced by loss of either histone deacetylases or the polycomb repressive complex 2 (Delgado-Olguín et al., 2012; He et al., 2012; Montgomery et al., 2007).

EXPERIMENTAL PROCEDURES

Animals

All animal work was carried out in accordance with the UK Animals (Scientific Procedures) Act 1986 and with the approval of the MRC National Institute of Medical Research Ethical Review Panel. Genotyping of the transgenic mouse lines *Nkx2-5-IRES-Cre* and *Nkx2-5-gfp* has been described previously (Prall et al., 2007).

ChIP Assay and Real-Time qPCR Analysis

Please refer to the Supplemental Experimental Procedures.

Sequencing and Peak Calling

Libraries were prepared with 10 ng immunoprecipitated DNA or input. Samples were sequenced on the Genome Analyzer IIx platform, and ChIP-seq reads were aligned to the mm9 genome assembly using BOWTIE. Peak calling was done using MACS1.4. Please refer to the Supplemental Experimental Procedures for more details.

Sequence and Gene Analysis

All enriched GO categories were determined using the programs GeneCoDis3 (Carmona-Saez et al., 2007) and GREAT for peak-associated genes. De novo motif identification was carried out using 50 bp around the summit of the peak of the top 1,033 regions using MEME-ChIP (Machanick and Bailey, 2011). Enhancer coordinates were found in the VISTA Enhancer Browser

(Visel et al., 2007) and compared to our set using Galaxy (Giardine et al., 2005). For comparison of the different ChIP-seq datasets, 10,000 random sets were generated and compared using pybedtools (Dale et al., 2011; Quinlan and Hall, 2010).

Motif distribution and overlap of the different NKX2-5 motifs among the identified peaks were done with FIMO using default parameters and the matrices identified with MEME.

Expression array data (GEO: GSE13614) used to analyze working myocardium genes versus atrio-ventricular genes were analyzed using Limma at Geo2r (http://www.ncbi.nlm.nih.gov/geo/geo2r/). The p values were adjusted for multiple testing using the Benjamin and Hochberg test, and transcripts were identified as differentially expressed if the adjusted p value was <0.01.

The region overlap between the NKX2-5 and MEIS dataset was determined using Galaxy (Goecks et al., 2010).

RNA-Seq

The E11.5 embryonic hearts from the hypomorphic NKX2-5 line were dissected and stored in RNAlater (QIAGEN). After genotyping, three control and three hypomorphic samples were selected for total RNA extraction using Trizol and purified on RNeasy columns (QIAGEN). Total RNA was converted to a library of templates suitable for high-throughput DNA sequencing using the Truseq kit (Illumina). Templates were sequenced using the Illumina Genome Analyzer IIx platform. Sequence reads were aligned to the mm9 genome with TopHat. Mapped reads were analyzed using the Avadis NGS package (Strand Scientific Intelligence; AC test fold change $\geq$1.5).

EMSA

Recombinant proteins were synthesized using a coupled transcription-translation system (Promega TnT T7/SP6 kit) and incubated with 0.5 ng radiolabelled oligonucleotide probe in binding buffer (20 mM Tris [pH 7.6], 75 mM KCl, 0.25 mg/ml, 1 mM DTT, and 10% glycerol) at room temperature for 10 min. For competition experiments, the excess of unlabelled oligonucleotide probes was added and incubated for 10 min prior to the addition of radiolabelled oligonucleotide probes. Binding reactions were analyzed on 6% 0.5× Tris-borate-EDTA (TBE) polyacrylamide gels electrophoresed at 150 V; gels were dried prior to detection of signal by autoradiography.

In Situ Hybridization

RNA in situ hybridizations were performed as described previously (Dupays et al., 2005). Tnnt3, Tnni2, and Lsp1 RNA probes were synthesized from whole IMAGE clones 3594256, 1448494, and 3488528, respectively.

Immunohistochemistry

Immunohistochemistry was performed as described previously (Dupays et al., 2009) with NKX2-5 (Santa Cruz Biotechnology) and MEIS1 (Abcam, ab19867) antibodies.

High-Resolution Episcopic Microscopy Imaging

Embryos were embedded and processed for imaging as described previously (Mohun and Weninger, 2011).

Transgenic Fish Experiments

Mouse DNA fragments were tested in vivo using the tol2 system (Allende et al., 2006; Fisher et al., 2006). Briefly, 1 kb amplified PCR product flanking the NKX2-5-enriched region was cloned in pCR8/GW/TOPO vector (Invitrogen) and subcloned into pGW_cfosGFP vector. Embryos were scored for GFP heart expression 48 hr after co-injection with Tol2 transposase into one-cell-stage zebrafish embryos.

Transgenic Mouse Experiments

The Popdc2 region tested previously in fish was subcloned in an hsp68-LacZ reporter gene. Transient transgenic mouse embryos were generated by the Procedural Services Section at The Francis Crick Institute Mill Hill Laboratory by standard pronuclear microinjection techniques.

ACCESSION NUMBERS

The accession number for the ChIP-seq and RNA-seq data reported in this paper is GEO: GSE44576.

SUPPLEMENTAL INFORMATION

Supplemental Information includes Supplemental Experimental Procedures, five figures, two tables, and four data files and can be found with this article online at http://dx.doi.org/10.1016/j.celrep.2015.08.065.

ACKNOWLEDGMENTS

We are indebted to Marie Caulfield and the Mill Hill Laboratory Biological Services for animal husbandry and technical support. We thank Abdul Sesay, Leena Bhaw-Rosun, and Harsha Jani at the Mill Hill Laboratory High Throughput DNA sequencing service for their technical help. Funding for this research was provided by the Medical Research Council (U117562103). This work was supported by the Francis Crick Institute, which receives its core funding from Cancer Research UK, the UK Medical Research Council, and the Wellcome Trust.

Received: March 5, 2015
Revised: June 17, 2015
Accepted: August 21, 2015
Published: September 24, 2015

REFERENCES

Akazawa, H., and Komuro, I. (2005). Cardiac transcription factor Csx/Nkx2-5: Its role in cardiac development and diseases. Pharmacol. Ther. *107*, 252–268.

Allende, M.L., Manzanares, M., Tena, J.J., Feijóo, C.G., and Gómez-Skarmeta, J.L. (2006). Cracking the genome's second code: enhancer detection by combined phylogenetic footprinting and transgenic fish and frog embryos. Methods *39*, 212–219.

Amin, S., Donaldson, I.J., Zannino, D.A., Hensman, J., Rattray, M., Losa, M., Spitz, F., Ladam, F., Sagerström, C., and Bobola, N. (2015). Hoxa2 selectively enhances Meis binding to change a branchial arch ground state. Dev. Cell *32*, 265–277.

Azcoitia, V., Aracil, M., Martínez-A, C., and Torres, M. (2005). The homeodomain protein Meis1 is essential for definitive hematopoiesis and vascular patterning in the mouse embryo. Dev. Biol. *280*, 307–320.

Biben, C., Weber, R., Kesteven, S., Stanley, E., McDonald, L., Elliott, D.A., Barnett, L., Köentgen, F., Robb, L., Feneley, M., and Harvey, R.P. (2000). Cardiac septal and valvular dysmorphogenesis in mice heterozygous for mutations in the homeobox gene Nkx2-5. Circ. Res. *87*, 888–895.

Blow, M.J., McCulley, D.J., Li, Z., Zhang, T., Akiyama, J.A., Holt, A., Plajzer-Frick, I., Shoukry, M., Wright, C., Chen, F., et al. (2010). ChIP-Seq identification of weakly conserved heart enhancers. Nat. Genet. *42*, 806–810.

Bradley, A., Anastassiadis, K., Ayadi, A., Battey, J.F., Bell, C., Birling, M.C., Bottomley, J., Brown, S.D., Bürger, A., Bult, C.J., et al. (2012). The mammalian gene function resource: the International Knockout Mouse Consortium. Mamm. Genome *23*, 580–586.

Briggs, L.E., Takeda, M., Cuadra, A.E., Wakimoto, H., Marks, M.H., Walker, A.J., Seki, T., Oh, S.P., Lu, J.T., Sumners, C., et al. (2008). Perinatal loss of Nkx2-5 results in rapid conduction and contraction defects. Circ. Res. *103*, 580–590.

Bruneau, B.G. (2008). The developmental genetics of congenital heart disease. Nature *451*, 943–948.

Cai, C.L., Liang, X., Shi, Y., Chu, P.H., Pfaff, S.L., Chen, J., and Evans, S. (2003). Isl1 identifies a cardiac progenitor population that proliferates prior to differentiation and contributes a majority of cells to the heart. Dev. Cell *5*, 877–889.

Cai, C.L., Zhou, W., Yang, L., Bu, L., Qyang, Y., Zhang, X., Li, X., Rosenfeld, M.G., Chen, J., and Evans, S. (2005). T-box genes coordinate regional rates of proliferation and regional specification during cardiogenesis. Development *132*, 2475–2487.

Carmona-Saez, P., Chagoyen, M., Tirado, F., Carazo, J.M., and Pascual-Montano, A. (2007). GENECODIS: a web-based tool for finding significant concurrent annotations in gene lists. Genome Biol. *8*, R3.

Dale, R.K., Pedersen, B.S., and Quinlan, A.R. (2011). Pybedtools: a flexible Python library for manipulating genomic datasets and annotations. Bioinformatics *27*, 3423–3424.

Delgado-Olguín, P., Huang, Y., Li, X., Christodoulou, D., Seidman, C.E., Seidman, J.G., Tarakhovsky, A., and Bruneau, B.G. (2012). Epigenetic repression of cardiac progenitor gene expression by Ezh2 is required for postnatal cardiac homeostasis. Nat. Genet. *44*, 343–347.

Domian, I.J., Chiravuri, M., van der Meer, P., Feinberg, A.W., Shi, X., Shao, Y., Wu, S.M., Parker, K.K., and Chien, K.R. (2009). Generation of functional ventricular heart muscle from mouse ventricular progenitor cells. Science *326*, 426–429.

Dupays, L., Jarry-Guichard, T., Mazurais, D., Calmels, T., Izumo, S., Gros, D., and Théveniau-Ruissy, M. (2005). Dysregulation of connexins and inactivation of NFATc1 in the cardiovascular system of Nkx2-5 null mutants. J. Mol. Cell. Cardiol. *38*, 787–798.

Dupays, L., Kotecha, S., Angst, B., and Mohun, T.J. (2009). Tbx2 misexpression impairs deployment of second heart field derived progenitor cells to the arterial pole of the embryonic heart. Dev. Biol. *333*, 121–131.

Fisher, S., Grice, E.A., Vinton, R.M., Bessling, S.L., Urasaki, A., Kawakami, K., and McCallion, A.S. (2006). Evaluating the biological relevance of putative enhancers using Tol2 transposon-mediated transgenesis in zebrafish. Nat. Protoc. *1*, 1297–1305.

Froese, A., and Brand, T. (2008). Expression pattern of Popdc2 during mouse embryogenesis and in the adult. Dev. Dyn. *237*, 780–787.

Giardine, B., Riemer, C., Hardison, R.C., Burhans, R., Elnitski, L., Shah, P., Zhang, Y., Blankenberg, D., Albert, I., Taylor, J., et al. (2005). Galaxy: a platform for interactive large-scale genome analysis. Genome Res. *15*, 1451–1455.

Goecks, J., Nekrutenko, A., and Taylor, J.; Galaxy Team (2010). Galaxy: a comprehensive approach for supporting accessible, reproducible, and transparent computational research in the life sciences. Genome Biol. *11*, R86.

He, A., Kong, S.W., Ma, Q., and Pu, W.T. (2011). Co-occupancy by multiple cardiac transcription factors identifies transcriptional enhancers active in heart. Proc. Natl. Acad. Sci. USA *108*, 5632–5637.

He, A., Ma, Q., Cao, J., von Gise, A., Zhou, P., Xie, H., Zhang, B., Hsing, M., Christodoulou, D.C., Cahan, P., et al. (2012). Polycomb repressive complex 2 regulates normal development of the mouse heart. Circ. Res. *110*, 406–415.

Hisa, T., Spence, S.E., Rachel, R.A., Fujita, M., Nakamura, T., Ward, J.M., Devor-Henneman, D.E., Saiki, Y., Kutsuna, H., Tessarollo, L., et al. (2004). Hematopoietic, angiogenic and eye defects in Meis1 mutant animals. EMBO J. *23*, 450–459.

Hoffman, J.I., and Kaplan, S. (2002). The incidence of congenital heart disease. J. Am. Coll. Cardiol. *39*, 1890–1900.

Horsthuis, T., Buermans, H.P., Brons, J.F., Verkerk, A.O., Bakker, M.L., Wakker, V., Clout, D.E., Moorman, A.F., ’t Hoen, P.A., and Christoffels, V.M. (2009). Gene expression profiling of the forming atrioventricular node using a novel tbx3-based node-specific transgenic reporter. Circ. Res. *105*, 61–69.

Huang, Q.Q., Feng, H.Z., Liu, J., Du, J., Stull, L.B., Moravec, C.S., Huang, X., and Jin, J.P. (2008). Co-expression of skeletal and cardiac troponin T decreases mouse cardiac function. Am. J. Physiol. Cell Physiol. *294*, C213–C222.

Jay, P.Y., Harris, B.S., Maguire, C.T., Buerger, A., Wakimoto, H., Tanaka, M., Kupershmidt, S., Roden, D.M., Schultheiss, T.M., O’Brien, T.X., et al. (2004). Nkx2-5 mutation causes anatomic hypoplasia of the cardiac conduction system. J. Clin. Invest. *113*, 1130–1137.

Kathiriya, I.S., Nora, E.P., and Bruneau, B.G. (2015). Investigating the transcriptional control of cardiovascular development. Circ. Res. *116*, 700–714.

Kelly, R.G. (2012). The second heart field. Curr. Top. Dev. Biol. *100*, 33–65.

Lescroart, F., Kelly, R.G., Le Garrec, J.F., Nicolas, J.F., Meilhac, S.M., and Buckingham, M. (2010). Clonal analysis reveals common lineage relationships between head muscles and second heart field derivatives in the mouse embryo. Development *137*, 3269–3279.

Machanick, P., and Bailey, T.L. (2011). MEME-ChIP: motif analysis of large DNA datasets. Bioinformatics *27*, 1696–1697.

Mahmoud, A.I., Kocabas, F., Muralidhar, S.A., Kimura, W., Koura, A.S., Thet, S., Porrello, E.R., and Sadek, H.A. (2013). Meis1 regulates postnatal cardiomyocyte cell cycle arrest. Nature *497*, 249–253.

Mohun, T.J., and Weninger, W.J. (2011). Imaging heart development using high-resolution episcopic microscopy. Curr. Opin. Genet. Dev. *21*, 573–578.

Montgomery, R.L., Davis, C.A., Potthoff, M.J., Haberland, M., Fielitz, J., Qi, X., Hill, J.A., Richardson, J.A., and Olson, E.N. (2007). Histone deacetylases 1 and 2 redundantly regulate cardiac morphogenesis, growth, and contractility. Genes Dev. *21*, 1790–1802.

Paige, S.L., Thomas, S., Stoick-Cooper, C.L., Wang, H., Maves, L., Sandstrom, R., Pabon, L., Reinecke, H., Pratt, G., Keller, G., et al. (2012). A temporal chromatin signature in human embryonic stem cells identifies regulators of cardiac development. Cell *151*, 221–232.

Parmacek, M.S., and Solaro, R.J. (2004). Biology of the troponin complex in cardiac myocytes. Prog. Cardiovasc. Dis. *47*, 159–176.

Penkov, D., Mateos San Martín, D., Fernandez-Díaz, L.C., Rosselló, C.A., Torroja, C., Sánchez-Cabo, F., Warnatz, H.J., Sultan, M., Yaspo, M.L., Gabrieli, A., et al. (2013). Analysis of the DNA-binding profile and function of TALE homeoproteins reveals their specialization and specific interactions with Hox genes/proteins. Cell Rep. *3*, 1321–1333.

Pfeufer, A., van Noord, C., Marciante, K.D., Arking, D.E., Larson, M.G., Smith, A.V., Tarasov, K.V., Müller, M., Sotoodehnia, N., Sinner, M.F., et al. (2010). Genome-wide association study of PR interval. Nat. Genet. *42*, 153–159.

Prall, O.W., Menon, M.K., Solloway, M.J., Watanabe, Y., Zaffran, S., Bajolle, F., Biben, C., McBride, J.J., Robertson, B.R., Chaulet, H., et al. (2007). An Nkx2-5/Bmp2/Smad1 negative feedback loop controls heart progenitor specification and proliferation. Cell *128*, 947–959.

Quinlan, A.R., and Hall, I.M. (2010). BEDTools: a flexible suite of utilities for comparing genomic features. Bioinformatics *26*, 841–842.

Sakabe, N.J., Aneas, I., Shen, T., Shokri, L., Park, S.Y., Bulyk, M.L., Evans, S.M., and Nobrega, M.A. (2012). Dual transcriptional activator and repressor roles of TBX20 regulate adult cardiac structure and function. Hum. Mol. Genet. *21*, 2194–2204.

Shen, T., Aneas, I., Sakabe, N., Dirschinger, R.J., Wang, G., Smemo, S., Westlund, J.M., Cheng, H., Dalton, N., Gu, Y., et al. (2011). Tbx20 regulates a genetic program essential to adult mouse cardiomyocyte function. J. Clin. Invest. *121*, 4640–4654.

Smith, J.G., Magnani, J.W., Palmer, C., Meng, Y.A., Soliman, E.Z., Musani, S.K., Kerr, K.F., Schnabel, R.B., Lubitz, S.A., Sotoodehnia, N., et al.; Candidate-gene Association Resource (CARe) Consortium (2011). Genome-wide association studies of the PR interval in African Americans. PLoS Genet. *7*, e1001304.

Stankunas, K., Shang, C., Twu, K.Y., Kao, S.C., Jenkins, N.A., Copeland, N.G., Sanyal, M., Selleri, L., Cleary, M.L., and Chang, C.P. (2008). Pbx/Meis deficiencies demonstrate multigenetic origins of congenital heart disease. Circ. Res. *103*, 702–709.

Stanley, E.G., Biben, C., Elefanty, A., Barnett, L., Koentgen, F., Robb, L., and Harvey, R.P. (2002). Efficient Cre-mediated deletion in cardiac progenitor cells conferred by a 3’UTR-ires-Cre allele of the homeobox gene Nkx2-5. Int. J. Dev. Biol. *46*, 431–439.

Tanaka, M., Chen, Z., Bartunkova, S., Yamasaki, N., and Izumo, S. (1999). The cardiac homeobox gene Csx/Nkx2.5 lies genetically upstream of

multiple genes essential for heart development. Development *126*, 1269–1280.

van den Berg, G., Abu-Issa, R., de Boer, B.A., Hutson, M.R., de Boer, P.A., Soufan, A.T., Ruijter, J.M., Kirby, M.L., van den Hoff, M.J., and Moorman, A.F. (2009). A caudal proliferating growth center contributes to both poles of the forming heart tube. Circ. Res. *104*, 179–188.

van den Boogaard, M., Wong, L.Y., Tessadori, F., Bakker, M.L., Dreizehnter, L.K., Wakker, V., Bezzina, C.R., 't Hoen, P.A., Bakkers, J., Barnett, P., and Christoffels, V.M. (2012). Genetic variation in T-box binding element functionally affects SCN5A/SCN10A enhancer. J. Clin. Invest. *122*, 2519–2530.

Visel, A., Minovitsky, S., Dubchak, I., and Pennacchio, L.A. (2007). VISTA Enhancer Browser–a database of tissue-specific human enhancers. Nucleic Acids Res. *35*, D88–D92.

Wamstad, J.A., Alexander, J.M., Truty, R.M., Shrikumar, A., Li, F., Eilertson, K.E., Ding, H., Wylie, J.N., Pico, A.R., Capra, J.A., et al. (2012). Dynamic and coordinated epigenetic regulation of developmental transitions in the cardiac lineage. Cell *151*, 206–220.

Watanabe, Y., Zaffran, S., Kuroiwa, A., Higuchi, H., Ogura, T., Harvey, R.P., Kelly, R.G., and Buckingham, M. (2012). Fibroblast growth factor 10 gene regulation in the second heart field by Tbx1, Nkx2-5, and Islet1 reveals a genetic switch for down-regulation in the myocardium. Proc. Natl. Acad. Sci. USA *109*, 18273–18280.

Cell Reports

Supplemental Information

Sequential Binding of MEIS1 and NKX2-5 on the Popdc2 Gene: A Mechanism for Spatiotemporal Regulation of Enhancers during Cardiogenesis

Laurent Dupays, Catherine Shang, Robert Wilson, Surendra Kotecha, Sophie Wood, Norma Towers, and Timothy Mohun

Supplemental Experimental Procedures

Chromatin Immunoprecipitation assay

Chromatin was prepared from 20-25 mouse embryonic hearts at stage E11.5. Tissue was fixed for 3 hours at room temperature in buffer (50mM HEPES pH7.9, 1mM EDTA, 1mM EGTA, 100mM NaCl, 0.07% butryric acid) containing 1.8% formaldehyde. Heart tissue was homogenised using an Ultra-Turrax, T25 basic (IKA-Werke), and pelleted at low speed. Samples were washed twice with ice-cold PBS with freshly added EDTA-free protease inhibitors (PI) (Roche). Samples were resuspended in 1.5ml lysis buffer (25mM Tris pH 7.5, 150mM NaCl, 1% Triton X100, 1% SDS, 2.5mM Sodium deoxycholate, PI) and transferred to RNase-free non-stick microfuge tubes (Ambion). Samples sonicated for 15 x 30 seconds on ice using a Branson Digital Sonicator with a 2.5mm stepped probe tip. Samples were spun at 15K using a bench top centrifuge for 15 minutes at 4oC. The supernatant was then pre-blocked with protein A/G sepharose (Perbio), pre-treated with BSA (Biorad) and Poly (dI-dC)-poly (dI-dC) (GE Healthcare). A proportion of the sample was kept at this stage as an input control. 150ml of chromatin was diluted 10 fold in ChIP dilution buffer (16.7mM Tris pH7.5, 0.01% SDS, 1.1% Triton, 1.2mM EDTA, 167mM NaCl) and 4mg of the antibody was added and incubated overnight at 4°C with rotation. The antibody:protein:DNA complexes were captured using pre-blocked protein A/G sepharose for 2 hours rotating at 4°C. The beads were then spun down and washed twice in wash buffer A (10mM HEPES pH 7.6, 1mM EDTA, 0.5mM EGTA, 0.25% Triton X100)

and twice with wash buffer B (10mM HEPES pH7.6, 200mM NaCl, 1mM EDTA, 0.5mM EGTA, 0.01% Triton X100. After extensive washing the beads were resuspended in TE and cross-links reversed overnight at 65°C. Samples were adjusted to 0.1% SDS, digested with 10mg Proteinase K (Roche) at 50°C for 3 hours and extracted twice with phenol/chloroform. Samples were ethanol precipitated with glycogen carrier (Ambion), and resuspended in 100ml TE. Antibodies used in the ChIP assays were NKX-2.5 (N-19) sc-8697 (Santa Cruz) and MEIS1 (Abcam, ab19867).

For experiment at E9.5, 40 to 50 embryos were dissected and AHF, heart were separately collected and processed as described previously.

Quantitative Real-Time PCR analysis

The fold enrichment of genomic targets in the immunoprecipitated DNA was determined by Real-Time quantitative PCR (qPCR) using the ABI PRISM 7000 sequence detection system (Applied Biosystems). Regions were considered enriched in binding if they displayed greater than a cut-off value of 1.5-fold. Primers were designed using Primer Express software (Applied Biosystems) and are available on demand. Relative fold enrichment was determined by normalising the Ct values for input (ΔCt) by subtracting the average Ct value of input from average Ct value of IP ($\Delta Ct = averageCt_{ChIP} - averageCt_{input}$). Enrichment was then calculated relative to a negative control (promoter region of eye specific g-crystallin gene) using the formula $2\text{-}(\Delta Ct_{[target]} - \Delta Ct_{[gCrystallin]})$. Fold

enrichment was calculated by dividing the enrichment values from the antibody-specific- ChIP by the enrichment values obtained in the background (no antibody) control samples. All qPCR were performed on at least four independent ChIPs.

Sequencing and peak calling

Libraries were prepared according to Illumina's instructions accompanying the DNA Sample Kit (Part# 0801-0303) with 10ng of immunoprecipitated DNA or input. Samples were sequenced on the Genome Analyser IIx platform and initial analysis was carried out using Illumina's CASAVA software. ChIP-seq reads were aligned to the mm9 genome assembly using BOWTIE. Peak calling was done using MACS1.4 (Stringency: p-value = 1x10-14) on the two independent experiments (s1 and s4) using a background experiment consisting of the input DNA. 2610 peaks were identified as the intersection between peaks called in the s1 and s4 experiments. Gene to peak assignment was achieved using GREAT (Two nearest genes association rule) (McLean et al., 2010).

Cell culture and stable transfections

HL-1 cardiac myocytes were maintained as previously reported (Claycomb et al., 1998). Briefly, HL-1 cells were grown in Claycomb medium (Sigma-Aldrich) supplemented with 10% fetal bovine serum (Sigma-Aldrich), 0.1 mM norepinephrine (Sigma-Aldrich), 2 mM L-glutamine (Sigma-Aldrich), and penicillin/streptomycin (Invitrogen) in a humidified 5% CO_2 incubator at 37°C on

fibronectin-coated flasks. After several passages, cells were transfected with 1 mg of shRNA against *Nkx2-5* (SureSilencing shRNA Plasmid for Mouse *Nkx2-5*, KM04476P, Qiagen) per plate, (or with comparable amounts of a control) using Fugene reagent (Roche). On the second day after transfection, fresh growth media supplemented with 1 μg/ml puromycin (Invitrogen) as the selection agent was added. Selection was allowed to proceed for 14 days, and the resulting colonies were harvested and maintained in the selection medium.

Immunoblotting

Cells were homogenized in 0.5 ml of RIPA buffer (20 mM Tris-HCl, pH 8.0; 150 mM NaCl; 0.1% SDS; 1% NP-40; 0.5% Sodium deoxycholate; and complete protease inhibitors cocktail; Roche). Protein concentration was determined with the Pierce BCA protein assay kit. 20 mg of protein extracts were separated on SDS-polyacrylamide gels and transferred to nitrocellulose membranes. Membranes were probed with an antibody against NKX2-5 (1:500) and subsequently with a goat anti-mouse IgG-HRP (1:2,000 dilution, sc-2005; Santa Cruz Bio- technology Inc.).

Dual Luciferase Reporter Assay

Denovo were synthesize in vitro and clone into pGL3 promoter vector (Promega).

Tnni2-Tnnt3 481bp enhancer was amplified by PCR from genomic DNA with the primers forward: TGCTACTCTTGGGGCTGACTTTAT and reverse GGACACCTGCCTGCTTTTTCTTC and cloned into pGL3 promoter vector (Promega). Tnni2-Tnnt3 mutated enhancer was obtain using QuickChange II site directed mutagenesis kit (Agilant Technologies) with the primers Forward: 5' CAGTGAAAACCTCTTAAGCCTCGTCAGGACAGAAGTGGC 3' and Reverse: 5' GCCACTTCTGTCCTGACGAGGCTTAAGAGGTTTTCACTG 3'.

Meis1 and Nkx2-5 cDNA cloned into pcDNA3 were transfected accordingly to the quantity indicate on the figures. pcDNA3 alone was used as control.

Transient transfections were performed in HL-1 cells or 3T3 cells using Lipofectamine 2000 (Promega) according to the manufacturer's instructions. Total cell lysates were prepared 48 hr posttransfection and luciferase activity was assessed using the Promega Dual Luciferase Reporter kit (Promega) according to the manufacturer's protocol. Results were normalised to a renilla transfection control.

Claycomb, W.C., Lanson, N.A., Jr., Stallworth, B.S., Egeland, D.B., Delcarpio, J.B., Bahinski, A., and Izzo, N.J., Jr. (1998). HL-1 cells: a cardiac muscle cell line that contracts and retains phenotypic characteristics of the adult cardiomyocyte. Proc Natl Acad Sci U S A 95, 2979-2984.

McLean, C.Y., Bristor, D., Hiller, M., Clarke, S.L., Schaar, B.T., Lowe, C.B., Wenger, A.M., and Bejerano, G. (2010). GREAT improves functional interpretation of cis-regulatory regions. Nat Biotechnol 28, 495-501.

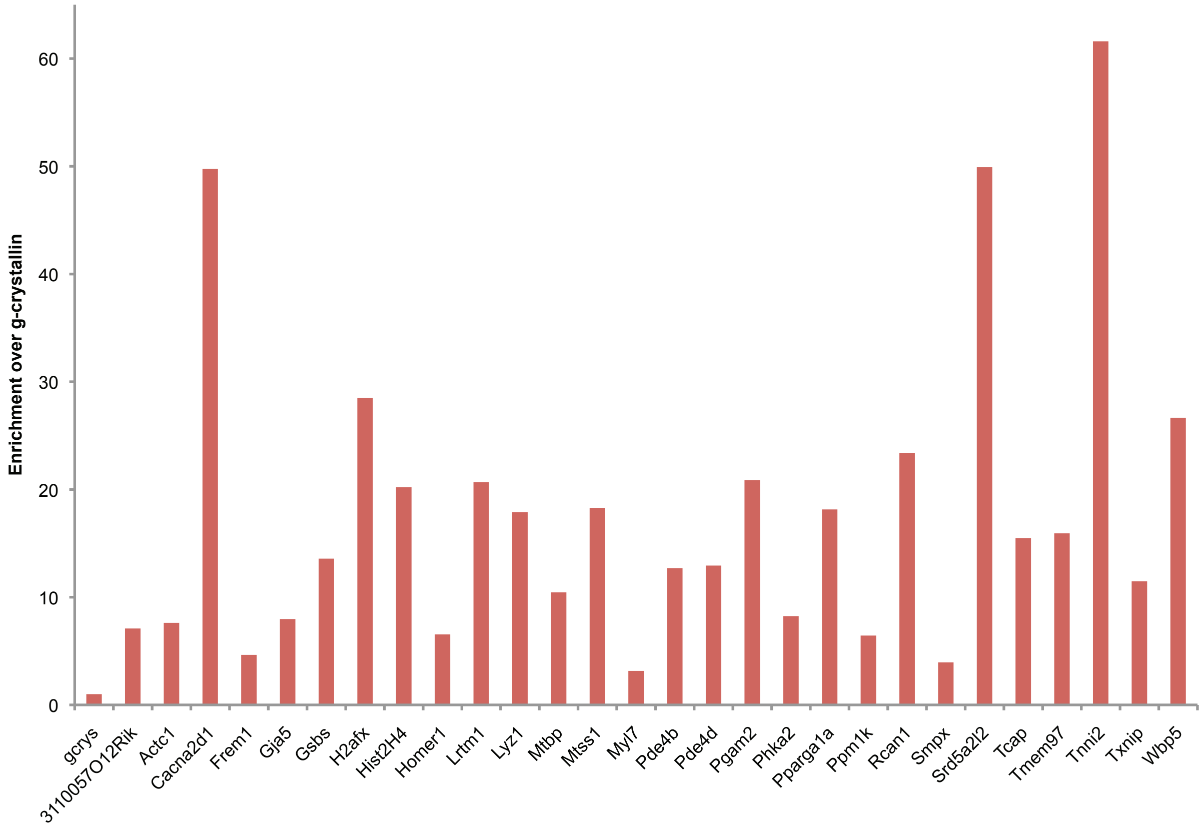

Figure S1. Validation of NKX2-5 enriched regions by ChIP-qPCR, Related to figure 1. Chromatin immunoprecipitation followed by quantitative PCR showing relative enrichment of the chosen enriched region for NKX2-5 binding over a negative control region in the gamma-crystallin gene using chromatin from E11.5 embryonic hearts.

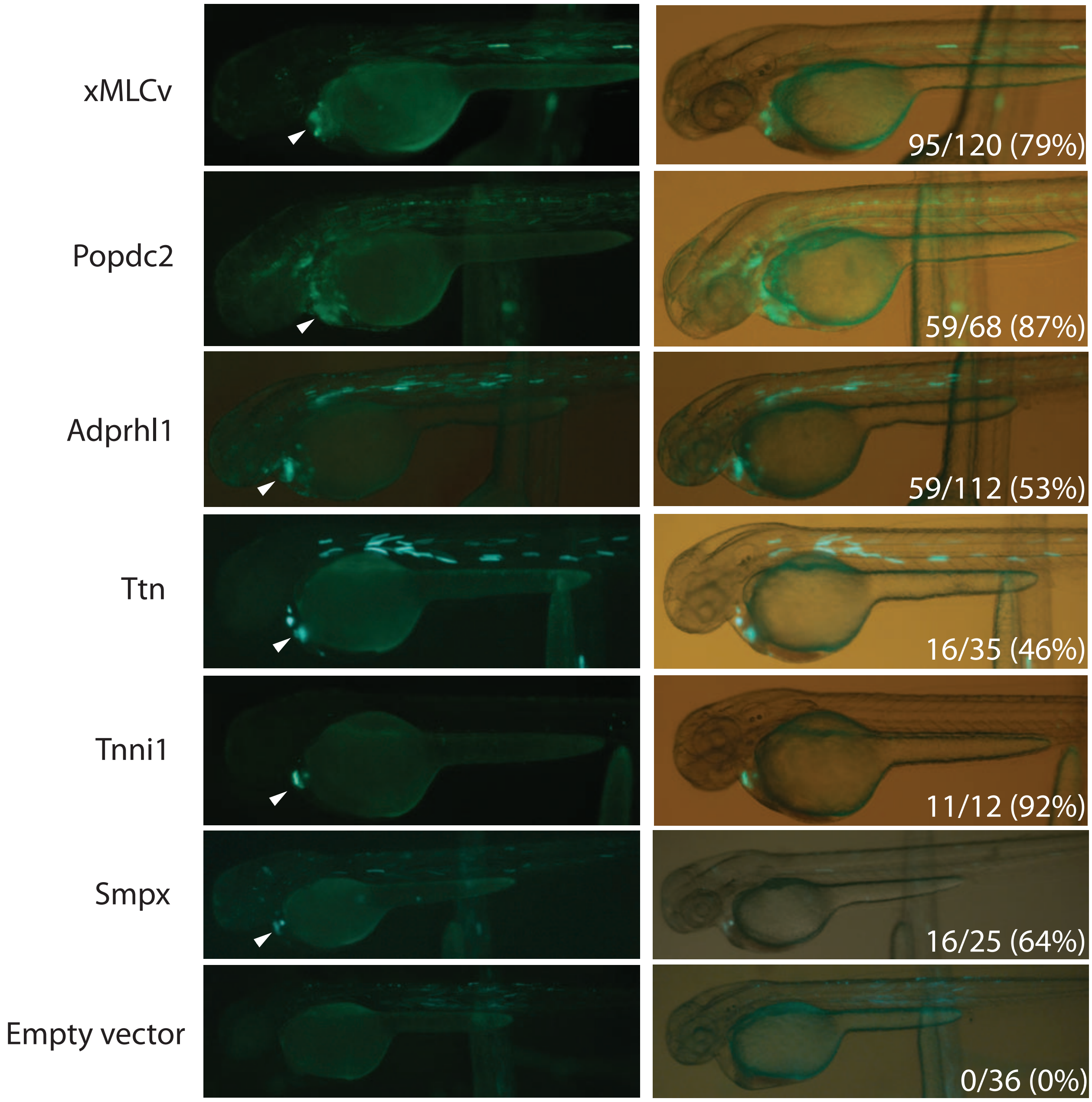

Figure S2. Validation of NKX2-5 enriched regions as enhancers, Related to Figure 1. Five different NKX2-5-binding regions (associated with the genes Popdc2, Adprhl1, Ttn, Tnni1 and Smpx) were tested for their ability to drive cardiac expression (white arrow-heads). Left side view of a representative transgenic zebrafish embryo at 48 hours post-fertilization (Left panel: GFP filter; right panel: overlay with bright field.). Percent-age of cardiac expressing embryos in the GFP positive pool is indicated. xMLC is used as a positive cardiac enhancer. Note that an empty vector shows no detectable GFP expression.

A

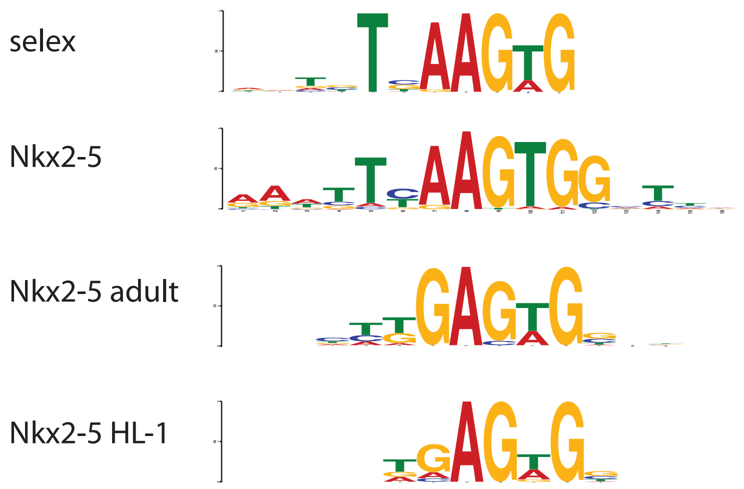

B

selex	TGCCCAGTCAAGTGTTCTTGA
denovo	ACAAAGTGGTGACAGGATCC
denovo m1	ACAAAGCCTCGACAGGATCC
denovo m2	ACAAAGTGGTCCTCGGATCC

Figure S3. ChIP-seq motif discovery comparison, Related to Figure 1. (A) Comparison of the selex NKX2-5 DNA binding motif with published binding motifs "NKX2-5" (Uniprobe), "NKX2-5 adult" (van den Boogaard et al., 2012) and "NKX2-5 HL-1" (He et al., 2011). (B) Sequence of the different probes used in the EMSA assay.

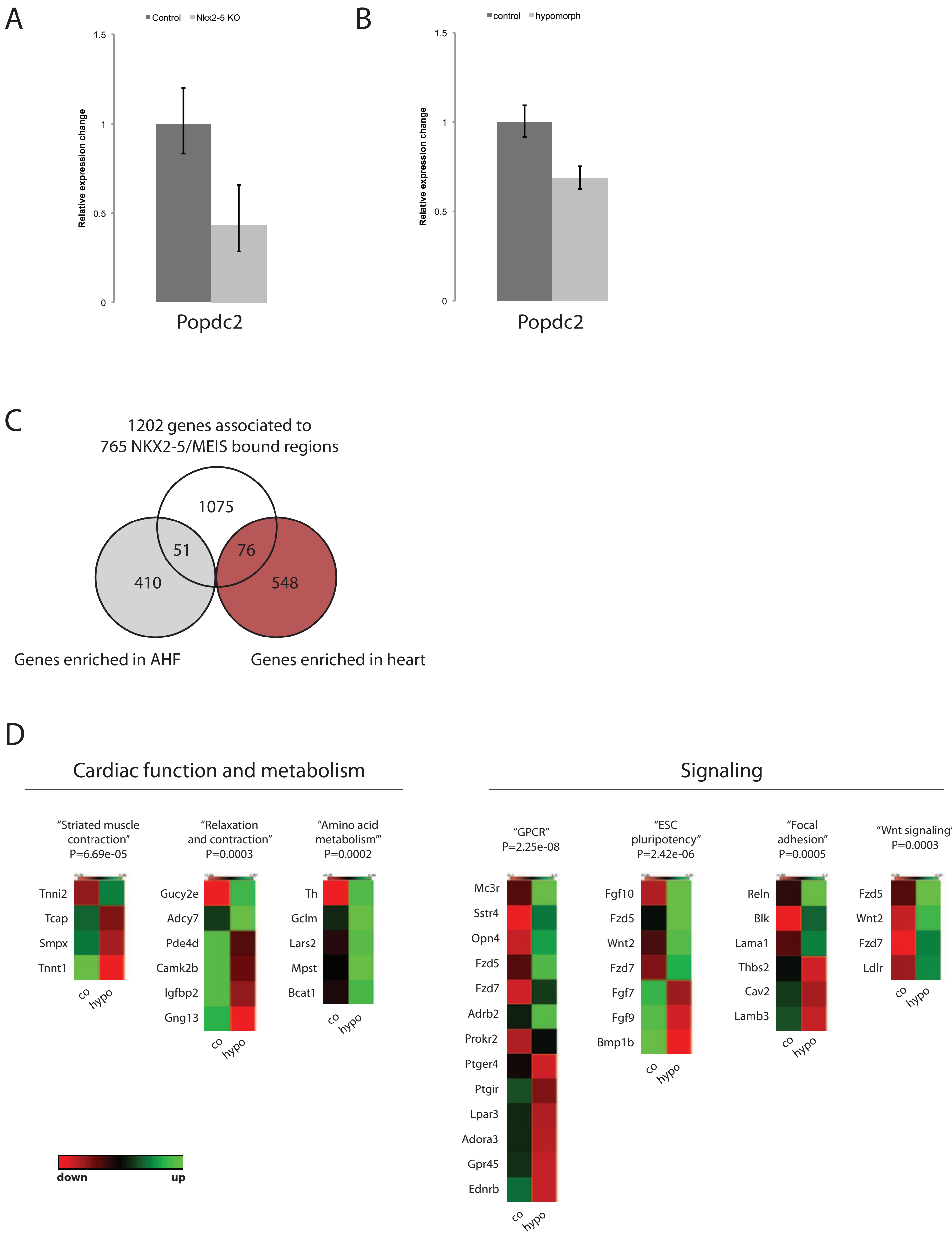

Figure S4. Enriched pathways, Related to Figure 5. Panels (A) and (B): Relative quantitative RT-PCR for Popdc2 mRNA expression in isolated embryo hearts. (A) Control and NKX2-5 heart knock out (Nkx2-5-gfp/Nkx2-5-gfp) at E9; (B) control and NKX2-5 hypomorph (Nkx2-5-IRES-cre/Nkx2-5-gfp) at E11.5. Panel C: Venn diagram showing the number of genes associated with an NKX2-5/MEIS bound region that are also enriched in AHF (51 genes) or heart (76 genes) according to Domian et al., 2009. Panel (D): The top seven Wikipathways enriched in the direct targets list. For each pathway, a p value is indicated. A heat map for each gene is shown, using data from RNA-seq with the NKX2-5 mutant hypomorph.

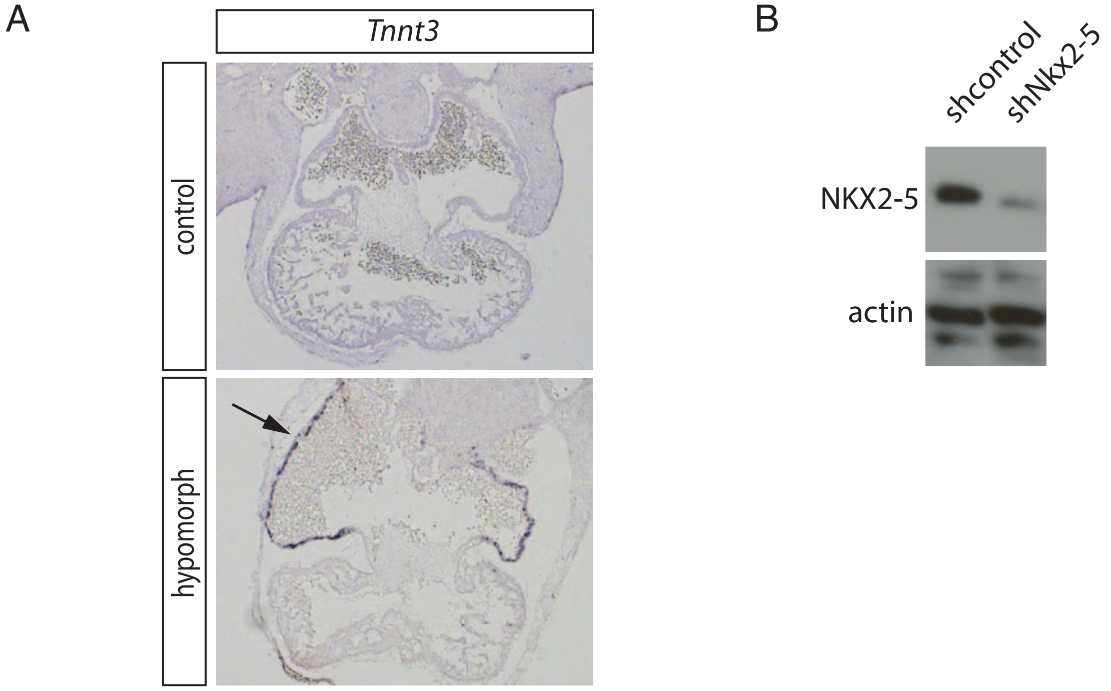

Figure S5. Tnnt3 expression at E11.5, Related to Figure 6. In situ hybridization on sections from E11.5 control and hypomorphic hearts, showing misexpression of Tnnt3 RNA in the atria (black arrow). (B) Western-blot showing downregulation of NKX2-5 protein expression in HL-1 cells transfected with a shRNA against Nkx2-5.

		Total number of peaks	Number of peaks overlapping With Nkx2-5 set	%	Median randomized	%	Fold enrichement
This study	**Nkx2-5 E11.5**	2610					
Blow et al., 2010.	**p300 E11.5**	3597	392	15.02%	5	0.19%	78.4
Shen et al., 2011.	**Tbx20 adult**	4012	354	13.56%	4	0.15%	88.5
Van Den Boogaard et al., 2012.	**Gata4 adult**	1756	231	8.85%	1	0.04%	231.0
	**Nkx2-5 adult**	6705	635	24.33%	6	0.23%	105.8
	**Tbx3 adult**	13242	416	15.94%	11	0.42%	37.8
He et al., 2011.	**Gata4 HL_1**	16753	803	30.77%	17	0.65%	47.2
	**Mef2a HL-1**	1337	104	3.98%	1	0.04%	104.0
	**Nkx2-5 HL-1**	20573	638	24.44%	20	0.77%	31.9
	**Srf HL-1**	23806	506	19.39%	23	0.88%	22.0
	**Tbx5 HL-1**	55872	933	35.75%	59	2.26%	15.8
	**p300 HL-1**	1491	264	10.11%	1	0.04%	264.0
Amin et al., 2015.	**Meis 1st BA**	64407	765	29.31%	15	0.57%	51.0
	**Meis 2nd BA**	62628	787	30.15%	16	0.61%	49.2

Table S1. Overlap between E11.5 NKX2-5 enriched regions (this study) and previously published data for various cardiac transcription factors, Related to Figure 1. The number of regions enriched and the number of overlapping regions with each of these factors and NKX2-5 are presented. Median randomized region number represents the median of the distribution of randomized intersections (10.000 iterations) between each set and randomly generated set similar to NKX2-5. Fold enrichment represents the actual count divided by the median for each factor and can be considered as a score.

	+/+	gfp/+	cre/+	gfp / cre	Total number of embryos
E8.5	23.1%	19.2%	30.8%	26.9%	26
E9.5	27.8%	21.1%	22.2%	28.9%	90
E11.5	24.3%	27.8%	24.3%	23.6%	403
E12.5	31.0%	17.2%	24.1%	27.6%	58
E13.5	25.6%	23.1%	23.1%	28.2%	39
E14.5	22.6%	38.7%	21.0%	17.7%	62
Weaning	31.8%	36.4%	31.8%	0.0%	298

Table S2. Mendelian analysis, Related to Figure 5. Number of different embryos collected prenatally and postnatally using the hypomorphic transgenic line.
